# Ethyl Lactate Ameliorates Hepatic Steatosis and Acute‐on‐Chronic Liver Injury in Alcohol‐Associated Liver Disease by Inducing Fibroblast Growth Factor 21

**DOI:** 10.1002/advs.202409516

**Published:** 2024-12-11

**Authors:** Yang Jiang, Shuang Wei, Shiming Shen, Yuxiao Liu, Weitong Su, Dong Ding, Zengpeng Zheng, Haokai Yu, Tingting Zhang, Qiuli Yang, Jiuxiang Zhao, Yi Shen, Xia Fang, Liangcai Lin, Dongguang Xiao, Aoyuan Cui, Qin Wan, Yadong Zhang, Yu Li, Cuiying Zhang

**Affiliations:** ^1^ State Key Laboratory of Food Nutrition and Safety College of Biotechnology Tianjin University of Science and Technology Tianjin 300457 China; ^2^ CAS Key Laboratory of Nutrition Metabolism and Food Safety Shanghai Institute of Nutrition and Health University of Chinese Academy of Sciences Chinese Academy of Sciences Shanghai 200031 China; ^3^ CAS Engineering Laboratory for Nutrition Shanghai Institute of Nutrition and Health University of Chinese Academy of Sciences Chinese Academy of Sciences Shanghai 200031 China; ^4^ Sichuan Langjiu Co. Ltd Gulin Sichuan 646523 China; ^5^ Department of Endocrinology and Metabolism Metabolic Vascular Disease Key Laboratory of Sichuan Province The Affiliated Hospital of Southwest Medical University Luzhou Sichuan 646000 China

**Keywords:** alcohol‐associated liver disease, ethyl lactate, fibroblast growth factor 21, hepatic steatosis, lipogenesis

## Abstract

Aberrant upregulation of hepatic lipogenesis induced by chronic and excessive alcohol consumption is a critical driver of the progression of alcohol‐associated liver disease (ALD), however, no effective approaches inhibiting lipogenesis are currently available for treating ALD patients. Moreover, little is known about whether and how nonethanol ingredients in alcoholic beverages regulate the pathogenesis of ALD. Here the discovery of a small molecule that activates the production and secretion of fibroblast growth factor 21 (FGF21) is reported. It is shown that the activator ethyl lactate, a nonethanol ingredient found in distilled liquors, ameliorates alcoholic hepatosteatosis, inflammation and acute‐on‐chronic liver injury by stimulating FGF21. In response to chronic‐plus‐binge ethanol feeding or fasting, ethyl lactate mimics lipogenesis lowering effects by stimulating FGF21 production through the NAD^+^‐dependent deacetylase sirtuin 1 (SIRT1) signaling pathway. These ethyl lactate‐mediated beneficial effects are abolished by inhibition of SIRT1 through injection of EX527. Importantly, FGF21 deficiency in hepatocytes blocks the downregulation of lipogenesis by ethyl lactate and exacerbates alcoholic steatosis, inflammation and liver injury. The regulatory mechanism is discussed during the pathophysiological conditions and suggests new lines of research into the therapeutic use of a foodborne small molecule ethyl lactate.

## Graphical abstract

This study discovers a foodborne molecule ethyl lactate, as a nonethanol ingredient found in distilled liquor, which ameliorates alcohol‐associated steatohepatitis. Mechanistically, ethyl lactate promotes the production and secretion of FGF21 via SIRT1 signaling, thereby inhibiting mTOCR1 activity to reduce alcohol‐induced lipogenesis. The development of small molecule drugs of ethyl lactate or its analogs may offer promising therapies for treating ALD.

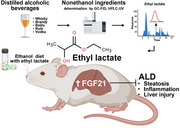

## 1 Introduction

Increasing global alcohol exposure exacerbates the worldwide prevalence of alcohol‐associated liver disease (ALD),^[^ ^1^ ^]^ which is a major cause of liver‐related morbidity and mortality.^[^ ^2^ ^]^ ALD encompasses a broad spectrum ranging from simple steatosis to alcohol‐associated steatohepatitis (ASH), fibrosis, cirrhosis, and hepatocellular carcinoma.^[^ ^2^, ^3^, ^4^, ^5^ ^]^ Alcohol‐associated steatotic liver (ASL), which is characterized by new lipid biosynthesis and accumulation, develops in over 90% of heavy drinkers and progresses to ASH, accompanied by hepatocyte damage and systemic inflammation, with limited therapeutic options available.^[^ ^4^, ^6^ ^]^ Thus, the development of novel therapeutic drugs for treating ALD is urgently needed.

Hepatic lipid metabolism encompasses a series of complex processes that control the influx and efflux of lipid pools within the liver.^[^ ^7^ ^]^ *De novo* lipogenesis, which provides newly synthesized fatty acids, is activated by alcohol consumption, and increased lipogenesis is the key feature associated with ASL.^[^ ^4^ ^]^ Hepatic lipogenesis is regulated by a critical transcription factor sterol regulatory element‐binding protein‐1 (SREBP‐1), which controls transcription of the rate‐limiting lipogenic enzymes, such as acetyl‐CoA carboxylase (ACC), fatty acid synthase (FAS) and stearoyl‐CoA‐desaturase 1 (SCD1).^[^ ^8^ ^]^ In mice and humans with ALD, the mechanistic target of rapamycin complex 1 (mTORC1) is activated by alcohol and promotes lipogenesis via SREBP‐1, whereas NAD^+^‐dependent deacetylase sirtuin 1 (SIRT1) is inhibited by alcohol.^[^ ^9^, ^10^ ^]^ Hepatic deficiency of SIRT1 enhanced mTORC1 activation and exacerbated the development of alcoholic fatty liver in mice.^[^ ^9^ ^]^ Considering that lipid accumulation is a prerequisite for the development of ALD, inhibiting hepatic fatty acid synthesis through multiple potential regulatory targets may offer a promising therapeutic strategy for treating ALD.^[^ ^11^ ^]^

The severity of ALD is highly dependent on the amount of alcohol intake, while the drinking patterns and types of alcoholic beverages contribute to the heterogeneity of ALD.^[^ ^12^ ^]^ The alcoholic beverage is a complex mixture of water, ethanol as well as a number of nonethanol chemical compounds. Compared with wine and beer, distilled spirits have higher ethanol content, which likely increases the susceptibility to more ethanol ingestion and ALD.^[^ ^4^ ^]^ Moreover, the nonethanol ingredients in alcoholic beverages are also especially enriched in distilled spirits. The nonethanol components in distilled spirits, largely falling into the categories of esters, alcohols, acids, and aldehydes, are primarily derived from the raw materials, processes of fermentation, distillation, and aging, which contribute to the unique flavor and taste of liquors.^[^ ^13^, ^14^, ^15^ ^]^ Interestingly, a clinical study of 68 nondrinkers or moderate social drinkers revealed that the nonethanol ingredients in whisky, such as aldehydes and fusel oils, promoted hangover symptoms,^[^ ^16^ ^]^ suggesting that these nonethanol ingredients may also be involved in regulating alcohol metabolism and related biological processes. However, whether nonethanol ingredients in distilled liquors regulate the pathogenesis of ALD remains largely unknown.

Here, using the mouse model of chronic‐plus‐binge alcohol feeding, which recapitulates the pathological features of human ALD, we screen the main nonethanol ingredients in spirits and reveal the function of ethyl lactate during ALD progression. These in vivo and in vitro studies demonstrate that 1) Ethyl lactate reduces susceptibility to developing fasting‐ and alcohol‐induced fatty liver, 2) Hepatic FGF21 is required for ethyl lactate‐caused amelioration of hepatic steatosis, inflammation and acute‐on‐chronic liver injury in chronic‐binge ethanol‐fed mice, 3) Defective FGF21 caused by pharmacologic inhibition of SIRT1 disrupts effects of ethyl lactate on alcohol‐induced hepatic steatosis, inflammation and liver injury in mice, 4) Ethyl lactate is a nonethanol ingredient of distilled liquors containing the inhibitory effect on alcohol‐induced lipogenesis. The current study identifies a biochemical mechanism of the nonethanol ingredient ethyl lactate in regulating FGF21 and reveals the functional importance of this signaling in the pathogenesis of alcoholic fatty liver diseases. Ethyl lactate is widely present not only in alcoholic beverages but also in other fermented foods. It is a recognized food flavoring agent and, as a dietary chemical compound, may hold therapeutic potential for treating alcoholic liver disease and other metabolic disorders.

## 2 Results

### 2.1 The Chemical Composition Signature of Nonethanol Ingredients in the Distilled Liquor

**Figure 1:**
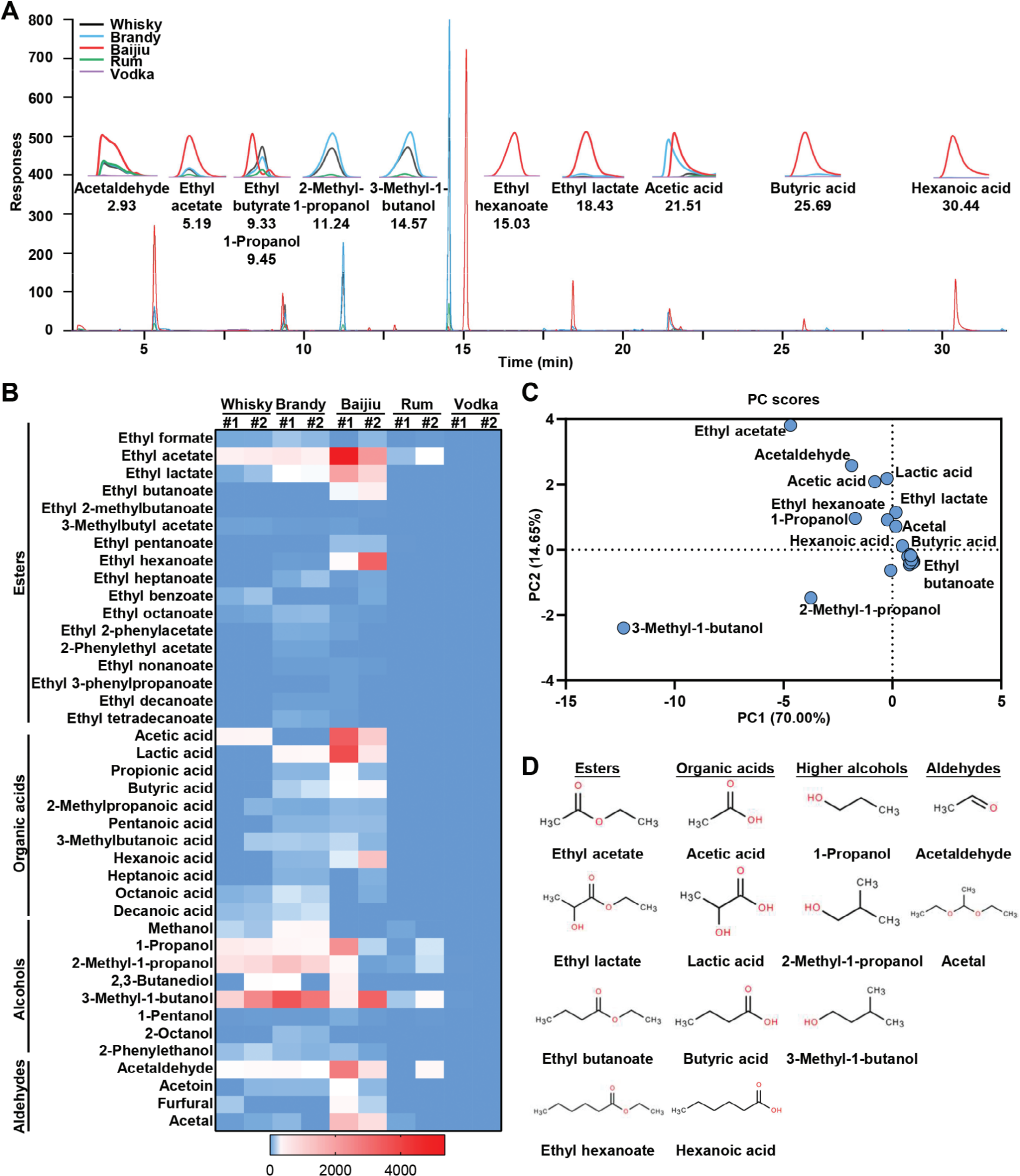
Characterization of the chemical composition signature of the nonethanol ingredients in distilled liquors. A) Representative GC‐FID spectra of chemical compounds in Whisky, Brandy, Baijiu, Rum, and Vodka, the principal strong peaks were enlarged. B) Heatmap analysis of concentration (in 100% ethanol) of compounds in 5 types of distilled spirits. n = 2. C) PCA analysis of chemistry in Whisky, Brandy, Baijiu, and Rum. n = 2/group. D) Chemical structures of main nonethanol ingredients detected in this study in distilled liquors.

To quantitatively determine the principal nonethanol ingredient in distilled liquors, gas chromatography with flame ionization detection (GC‐FID) and high‐performance liquid chromatography with UV detection (HPLC‐UV) were used.^[^ ^17^ ^]^ The representative GC‐FID spectra of the five typical distilled spirits, including Whisky, Brandy, Baijiu, Rum, and Vodka, were presented in **Figure** **1**A, where the principal strong peaks were enlarged for clarity. The concentrations of 40 chemicals (normalized to 100% ethanol) were shown in Table S1 (Supporting Information), and the heatmap revealed a notable diversity and variation in the composition of compounds across five distilled spirits (Figure 1B). Whisky and Brandy contain a higher concentration of higher alcohols, including 1‐propanol, 2‐methyl‐1‐propanol, and 3‐methyl‐1‐butanol, which originate from the fermentation process involving yeast. Baijiu, the most representative Chinese liquor, stands out with its rich content of esters (such as ethyl acetate, ethyl lactate, ethyl butanoate, and ethyl hexanoate) and acids (including acetic acid, lactic acid, butyric acid, and hexanoic acid). This is attributed to its unique solid‐state fermentation process involving yeast and other microorganisms like lactic acid bacteria. In contrast, Rum and Vodka exhibit a purer composition, containing only a minimal quantity of higher alcohols, like 3‐methyl‐1‐butanol. Next, principal component analysis (PCA) was performed, and 13 compounds were ultimately defined as key nonethanol ingredients in distilled spirits (Figure 1C). The structures of these chemicals are shown in Figure 1D. Together, 13 principal nonethanol ingredients in distilled liquors, including ethyl esters, organic acids, higher alcohols, and aldehydes, were determined in five typical distilled spirits (**Table** **1**).

**Table 1:** Concentrations of the key nonethanol ingredients determined in distilled liquors.

Chemistry	Molecular Formula	Concentrations [mg L^−1^ in 100% ethanol]				
		Whisky	Brandy	Baijiu	Rum	Vodka
Ethyl acetate	C_4_H_8_O_2_	457.4 ± 58.68	501.57 ± 124	3757 ± 2249.88	84.44 ± 69.23	N/D
Ethyl lactate	C_5_H_10_O_3_	26.74 ± 15.05	104.57 ± 14.17	1469.64 ± 727.18	N/D	N/D
Ethyl butanoate	C_6_H_12_O_2_	N/D	N/D	276.32 ± 261.71	N/D	N/D
Ethyl hexanoate	C_8_H_16_O_2_	N/D	N/D	0.93 ± 1.32	N/D	N/D
Acetic acid	C_2_H_4_O_2_	8.39 ± 0.95	3.06 ± 0.31	3.21 ± 4.54	N/D	N/D
Lactic acid	C_3_H_6_O_3_	N/D	N/D	34.43 ± 0.53	0.1 ± 0.14	N/D
Butyric acid	C_4_H_8_O_2_	N/D	3.74 ± 1.17	1745.7 ± 2199.72	N/D	N/D
Hexanoic acid	C_6_H_12_O_2_	N/D	27.61 ± 16.17	8.9 ± 6.67	N/D	N/D
1‐Propanol	C_3_H_8_O	29.43 ± 12.73	N/D	6.86 ± 9.7	N/D	N/D
2‐Methyl‐1‐propanol	C_4_H_10_O	10.11 ± 2.47	29.91 ± 1.1	4.45 ± 3.2	N/D	N/D
3‐Methyl‐1‐butanol	C_5_H_12_O	N/D	16.23 ± 1.02	3.9 ± 5.51	N/D	N/D
Acetaldehyde	C_2_H_4_O	N/D	10.15 ± 0.29	N/D	N/D	N/D
Acetal	C_6_H_14_O_2_	N/D	4.34 ± 0.13	4.45 ± 3.2	N/D	N/D

All values are means (mg/L in 100% ethanol) ± standard deviation (SD).

N/D: not detected.

### 2.2 Hepatic Steatosis, Inflammation, and Acute‐on‐Chronic Liver Injury in Chronic‐Binge Ethanol‐Fed Mice are Ameliorated by Ethyl Lactate

**Figure 2:**
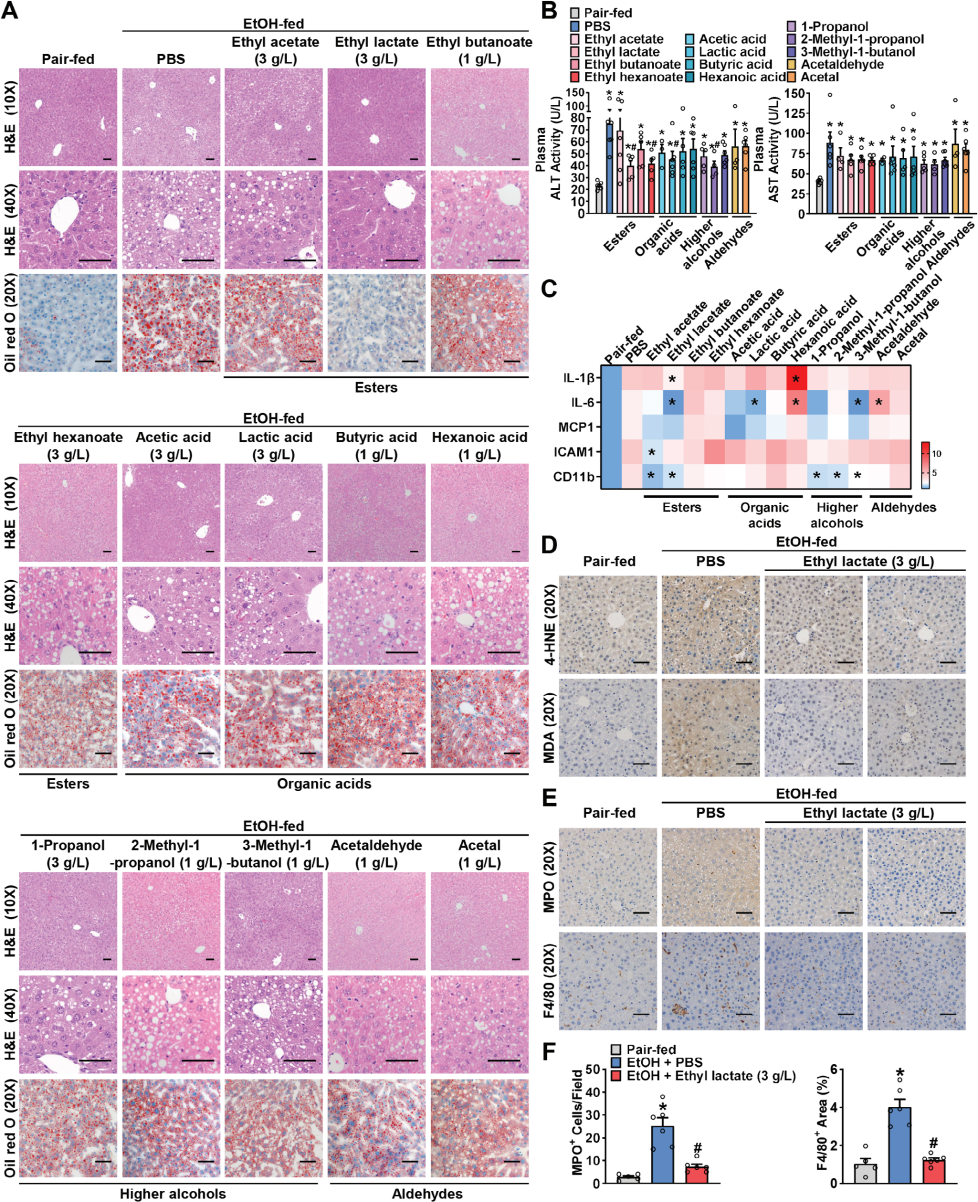
Ethyl lactate ameliorates hepatic steatosis, inflammation and acute‐on‐chronic liver injury in chronic‐binge ethanol‐fed mice. WT mice were fed a 5% (v/v) ethanol diet for 10 days, followed by gavage of ethanol (5 g kg^−1^), and were euthanized 9 h later (Gao‐Binge model). During ethanol feeding and gavage, nonethanol ingredients of the spirits were added in ethanol (52%) and then mixed in Liber–DeCarli diet. (Esters: ethyl acetate (3 g L^−1^ in 52% ethanol), ethyl lactate (3 g L^−1^), ethyl butanoate (1 g L^−1^), ethyl hexanoate (3 g L^−1^), Organic acids: acetic acid (3 g L^−1^), lactic acid (3 g L^−1^), butyric acid (1 g L^−1^), hexanoic acid (1 g L^−1^), Higher alcohols: 1‐propanol (3 g L^−1^), 2‐methyl‐1‐propanol (1 g L^−1^), 3‐methyl‐1‐butanol (1 g L^−1^), Aldehydes: acetaldehyde (1 g L^−1^), acetal (1 g L^−1^)). A–E) Compounds screening revealed that ethyl lactate as a potential chemistry regulating ASH. (A) Representative H&E and Oil red O staining in the liver sections of mice (scale bars: 50 µm). (B) Plasma ALT and AST activity levels. n = 3–6. ^*^ *p* < 0.05 versus pair‐fed mice, #*p* < 0.05 versus ethanol‐fed mice treated with PBS. (C) Heatmap of expression levels of inflammatory genes in mice livers. The mRNA levels were measured by real‐time PCR. n = 3–6. ^*^ *p* < 0.05 versus ethanol‐fed mice treated with PBS. (D‐E) Representative IHC staining with antibody against 4‐HNE, MDA (D), MPO and F4/80 (E). Scale bars: 50 µm. F) Quantification of number of MPO^+^ cells and percentage of F4/80^+^ area. n = 3–6. ^*^ *p* < 0.05 versus pair‐fed mice, ^#^ *p* < 0.05 versus ethanol‐fed mice treated with PBS.

To investigate the roles of the 13 key nonethanol ingredients in the development of ALD, mice were subjected to a standard Gao‐Binge model, which closely resembles the pathogenesis of ASH in humans and mimics the drinking patterns observed in many patients with ALD.^[^ ^18^ ^]^ Each individual compound was added to a chronic‐plus‐binge ethanol diet at the highest concentrations in previous studies.^[^ ^15^, ^19^ ^]^ The effects of these nonethanol ingredients on alcohol‐induced hepatic steatosis, injury, oxidative stress, and inflammation, while maintaining equal alcohol intake, were then examined (**Figure** **2**A–C). After chronic‐plus‐binge alcohol feeding, the mice developed ASH, as evidenced by hepatic lipid accumulation shown through hematoxylin and eosin (H&E) and Oil red O staining (Figure 2A), as well as elevated plasma levels of alanine aminotransferase (ALT) and aspartate aminotransferase (AST) (Figure 2B). The expression levels of inflammatory genes (IL‐1β, IL‐6, MCP1, ICAM1, CD11b) in livers were upregulated by alcohol feeding (Figure 2C). Interestingly, among the treatment with the 13 compounds, ethyl lactate (C_5_H_10_O_3_) treatment (3 g L^−1^ in 52% ethanol) significantly ameliorated alcohol‐induced hepatic steatosis, injury, and inflammation without significant changes in body weight and epididymal white adipose tissue (eWAT) weight (Figure S1, Supporting Information). As a hydrolysate of ethyl lactate, lactic acid had limited beneficial effects on ALD progression, suggesting the improved effects of ethyl lactate may be independent of its hydrolysis to produce lactic acid.

Oxidative stress and inflammation, especially infiltration of neutrophils, have been considered as two critical features of ALD.^[^ ^4^ ^]^ To further validate the efficacy of ethyl lactate, immunohistochemistry staining of liver sections was performed. As shown in Figure 2D–F, compared with PBS‐treated mice, ethyl lactate‐treated mice exhibited reduced ROS production, as evidenced by malonaldehyde (MDA) and 4‐hydroxynonenal (4‐HNE) staining, as well as decreased infiltration of MPO^+^ neutrophils and F4/80^+^ macrophages in livers. Together, these data suggest that ethyl lactate acts as a promising candidate for protecting chronic‐plus‐binge ethanol feeding‐induced steatosis, oxidative stress, inflammation, and liver injury.

### 2.3 The Reduction of Hepatic Lipogenesis and Mitigation of Hepatic Steatosis in Chronic‐Binge Ethanol‐Fed Mice by Ethyl Lactate in a Dose‐Dependent Manner

**Figure 3:**
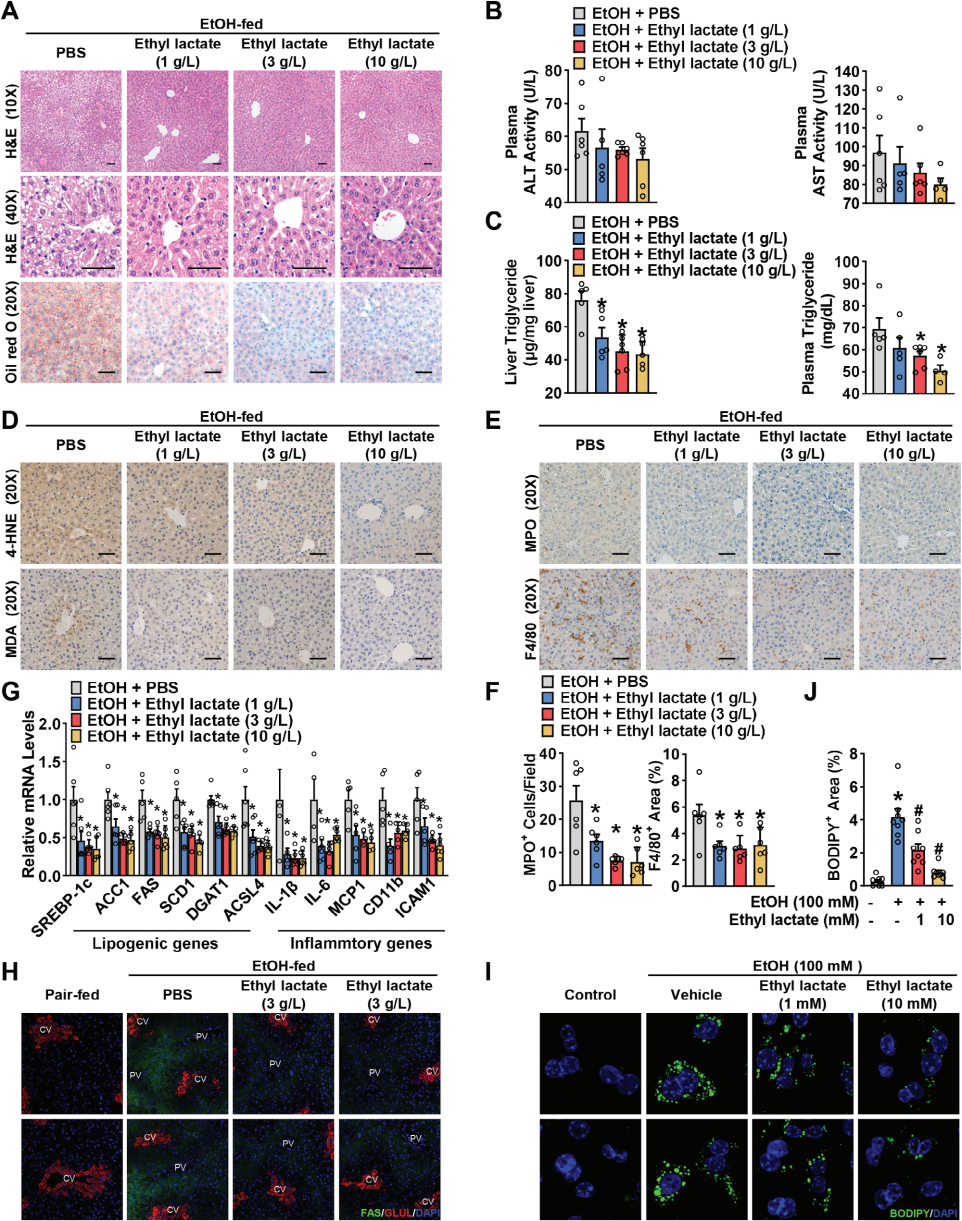
Ethyl lactate reduces hepatic lipogenesis and mitigates hepatic steatosis in chronic‐binge ethanol‐fed mice in a dose‐dependent manner. WT mice were subjected to chronic‐plus‐binge ethanol feeding, and a range dose of ethyl lactate (1, 3 or 10 g L^−1^ in 52% ethanol) in ethanol diet. A–G) Ethyl lactate improved hepatic steatosis, injury and inflammation in chronic‐binge ethanol fed‐mice. (A) Representative H&E and Oil red O staining in the liver sections of mice (Scale bars: 50 µm). (B) Plasma ALT and AST activity levels. (C) Hepatic triglyceride (TG) levels and plasma TG levels. (D‐E) Representative IHC staining with antibody against 4‐HNE, MDA (D), MPO and F4/80 (E) (Scale bars: 50 µm). (F) Quantification of number of MPO^+^ cells and percentage of F4/80^+^ area. (G) mRNA levels of lipogenic and inflammatory genes in the liver of mice. n = 4–6. ^*^ *p* < 0.05 versus ethanol‐fed mice treated with PBS. H) Representative immunofluorescent staining of FAS and GLUL in liver sections (20× magnification). I) Representative images of BODIPY staining of mouse primary hepatocytes treated with 100 mM ethanol and ethyl lactate for 48 h (63× magnification). J) Quantification of lipid droplets by BODIPY staining area. n = 8. ^*^ *p* < 0.05 versus control, ^#^ *p* < 0.05 versus ethanol.

To further validate the beneficial effects of ethyl lactate, ethyl lactate was initially added to 52% ethanol at concentrations of 1, 3, and 10 grams per liter, and then incorporated into a chronic‐plus‐binge ethanol diet (5% v/v). Consistently, as shown in **Figures** **3**A–C and S2 (Supporting Information), ethyl lactate treatment dose‐dependently decreased hepatic steatosis, liver injury, and liver and plasma triglyceride (TG) levels in ethanol‐fed mice. Notably, the body weight and food intake were not obviously changed. Furthermore, immunohistochemical analysis revealed that ethyl lactate reduced ROS production, infiltration of neutrophils and macrophages in the liver of mice treated at the low dose of 1 g L^−1^, and higher dose of ethyl lactate‐treated mice showed pronounced improvement of hepatic oxidative stress and inflammation (Figure 3D–F). Given that chronic alcohol feeding activates lipogenesis and leads to lipid accumulation, then the expression levels of key lipogenic genes in the liver were determined. Strikingly, ethyl lactate significantly reduced the mRNA levels of lipogenesis‐related genes, such as SREBP‐1c, ACC1, FAS, and SCD1 (Figure 3G). Consistently, ethanol‐fed mice administered a range of doses of ethyl lactate also exhibited lower expression of inflammatory genes. Interestingly, the alcohol‐induced elevation of lipogenic enzyme FAS was observed in periportal and midlobular hepatocytes, but not in pericentral hepatocytes labeled with glutamine synthetase (GLUL). Ethyl lactate treatment resulted in a profound suppression of FAS expression in the livers of mice fed with alcohol (Figure 3H).

Next, the significance of ethyl lactate in inhibiting alcohol‐induced lipogenesis was further determined. Primary hepatocytes were isolated from standard diet‐fed mice and incubated with 100 mM ethanol in the presence or absence of ethyl lactate for 48 h. Interestingly, compared with the vehicle control group, ethyl lactate significantly reduced the numbers and size of lipid droplets in hepatocytes exposed to ethanol, as indicated by BODIPY staining (Figure 3I–J). Taken together, these data suggest that ethyl lactate inhibits alcohol‐induced lipogenesis in hepatocytes and improves alcoholic liver diseases.

### 2.4 Ethyl Lactate Stimulates the Production and Secretion of FGF21 in Ethanol‐Treated Hepatocytes or in Livers of Chronic‐Binge Ethanol‐Fed Mice

**Figure 4:**
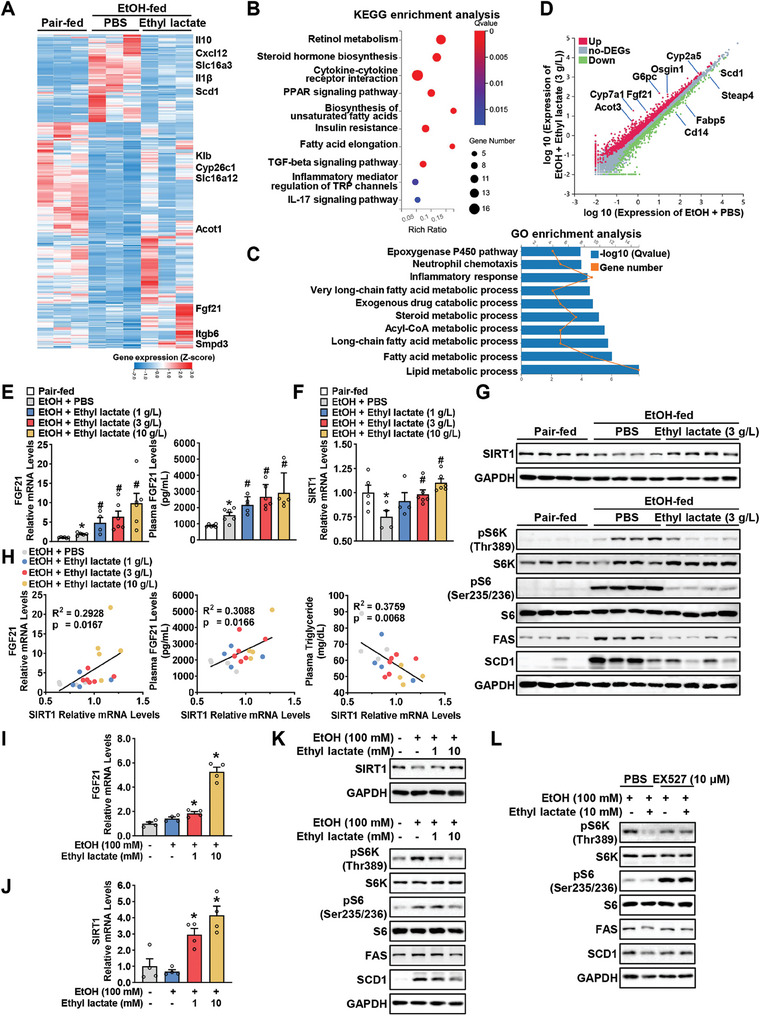
Production and secretion of FGF21 is induced by ethyl lactate in ethanol‐treated hepatocytes or in livers of chronic‐binge ethanol‐fed mice. WT mice were subjected to chronic‐plus‐binge ethanol feeding with/without a range dose of ethyl lactate (1, 3 or 10 g L^−1^ in 52% ethanol) treatment. A–D) RNA‐seq analysis of liver from pair‐fed and ethanol‐fed mice treated with PBS or ethyl lactate. n = 3/group. Heatmap (A), KEGG (B) and GO (C) analysis and scatter diagram (D) of significant differentially expressed 486 genes between ethanol‐fed mice with or without ethyl lactate treatment. E–F) Ethyl lactate enhanced hepatic and plasma FGF21 levels (E) as well as SIRT1 levels (F) in the liver of pair‐fed and ethanol‐fed mice, as determined by real‐time PCR and ELISA analysis. n = 4–6. ^*^ *p* < 0.05 versus pair‐fed, ^#^ *p* < 0.05 versus ethanol‐fed with PBS. G) Ethyl lactate increased protein levels of SIRT1 and decreased mTORC1 activities as well as the expression of lipogenesis proteins induced by ethanol‐feeding in livers of mice. n = 4/group. H) Correlation analyses were performed between mRNA levels of SIRT1 and FGF21, plasma FGF21 levels or plasma TG levels in ethanol‐fed mice. Data from Figure 4E,F and Figure 3C. I,J) Ethyl lactate enhanced FGF21 mRNA (I) and SIRT1 mRNA levels (J) in AML12 hepatocytes treated with 100 mM ethanol for 48 h. n = 4. ^*^ *p* < 0.05 versus ethanol treatment. K,L) Ethyl lactate increased protein levels of SIRT1, whereas inhibited alcohol‐induced mTORC1 activities and the expression of lipogenesis proteins (K), which were blocked by SIRT1 inhibitor EX527 in AML12 hepatocytes (L).

To investigate the underlying mechanisms by which ethyl lactate‐mediated improvement in hepatic lipogenesis, RNA‐sequencing was performed on the livers of mice that were pair‐fed or fed with ethanol, with or without ethyl lactate treatment. A total of 143 downregulated genes and 343 upregulated genes were identified in the liver of ethanol‐fed mice treated with ethyl lactate, compared with the control group (**Figure** **4**A). Among the significantly downregulated pathways identified through Gene Ontology (GO) and Kyoto Encyclopedia of Genes and Genomes (KEGG) analysis, the majority were involved in lipid metabolism and inflammation signaling, which are recognized as the key pathways in the pathogenesis of ALD (Figure 4B,C). The top differentially changed genes by ethyl lactate, including ACOT3, CYP7A1, FGF21, G6PC, OSGIN1, CYP2A5, and FABP5, were further verified by real‐time PCR (Figure 4E; Figure S3, Supporting Information). Compared with other genes, FGF21 is the gene that was dose‐dependently induced by ethyl lactate in the livers of ethanol‐fed mice. The secretion of FGF21 in plasma was also stimulated by ethyl lactate in ethanol‐fed mice (Figure 4E). FGF21 is a metabolic stress‐ and alcohol‐induced hormone,^[^ ^20^, ^21^, ^22^ ^]^ which is predominantly secreted by hepatocytes and functions as the metabolic regulator to maintain metabolic homeostasis and protect against ALD.^[^ ^23^, ^24^ ^]^ Administration of FGF21 has been shown to alleviate insulin resistance and hepatic lipogenesis by inhibiting mTORC1.^[^ ^25^ ^]^ Previously, SIRT1 deficiency in hepatocytes decreased hepatic and circulating levels of FGF21 under fasting conditions, suggesting the important roles of SIRT1 in regulating FGF21 in livers.^[^ ^26^ ^]^ Intriguingly, in the livers of ethanol‐fed mice, ethyl lactate increased the transcriptional and protein levels of SIRT1 (Figure 4F,G). Consistently, positive correlations were observed between hepatic SIRT1 levels with hepatic FGF21 expression and plasma FGF21 levels, whereas SIRT1 expression was negatively correlated with plasma TG levels in an ethyl lactate dose‐dependent manner (Figure 4H).

Alcohol‐induced hyperactivation of the mTORC1 drives hepatic lipogenesis and plays a key role in the pathogenesis of ALD.^[^ ^9^ ^]^ As shown in Figure 4G, ethyl lactate inhibited the ethanol‐caused activation of mTORC1, as evidenced by decreased phosphorylation of p70 S6 kinase (S6K) at Thr389 and its substrate S6 ribosomal protein (S6) at Ser235/Ser236 sites. Consistently, key lipogenic proteins FAS and SCD1 were also decreased by ethyl lactate treatment. The effects of ethyl lactate on the induction of FGF21 and inhibition of mTORC1 were further verified in AML12 hepatocytes. As shown in Figure 4I,K, ethyl lactate significantly increased expression levels of FGF21 and SIRT1 and suppressed the activity of mTORC1 signaling and lipogenic proteins in AML12 hepatocytes exposed to ethanol. However, these effects were abolished by SIRT1 inhibitor EX527^[^ ^27^ ^]^ (Figure 4L). Taken together, these results demonstrate that ethyl lactate inhibits alcohol‐induced lipogenesis and mTORC1 pathway via facilitating the expression and production of hepatocellular FGF21.

### 2.5 Ethyl Lactate Protects Against Fasting‐Induced Hepatic Steatosis in Mice by Stimulating the Production and Secretion of Hepatic FGF21

**Figure 5:**
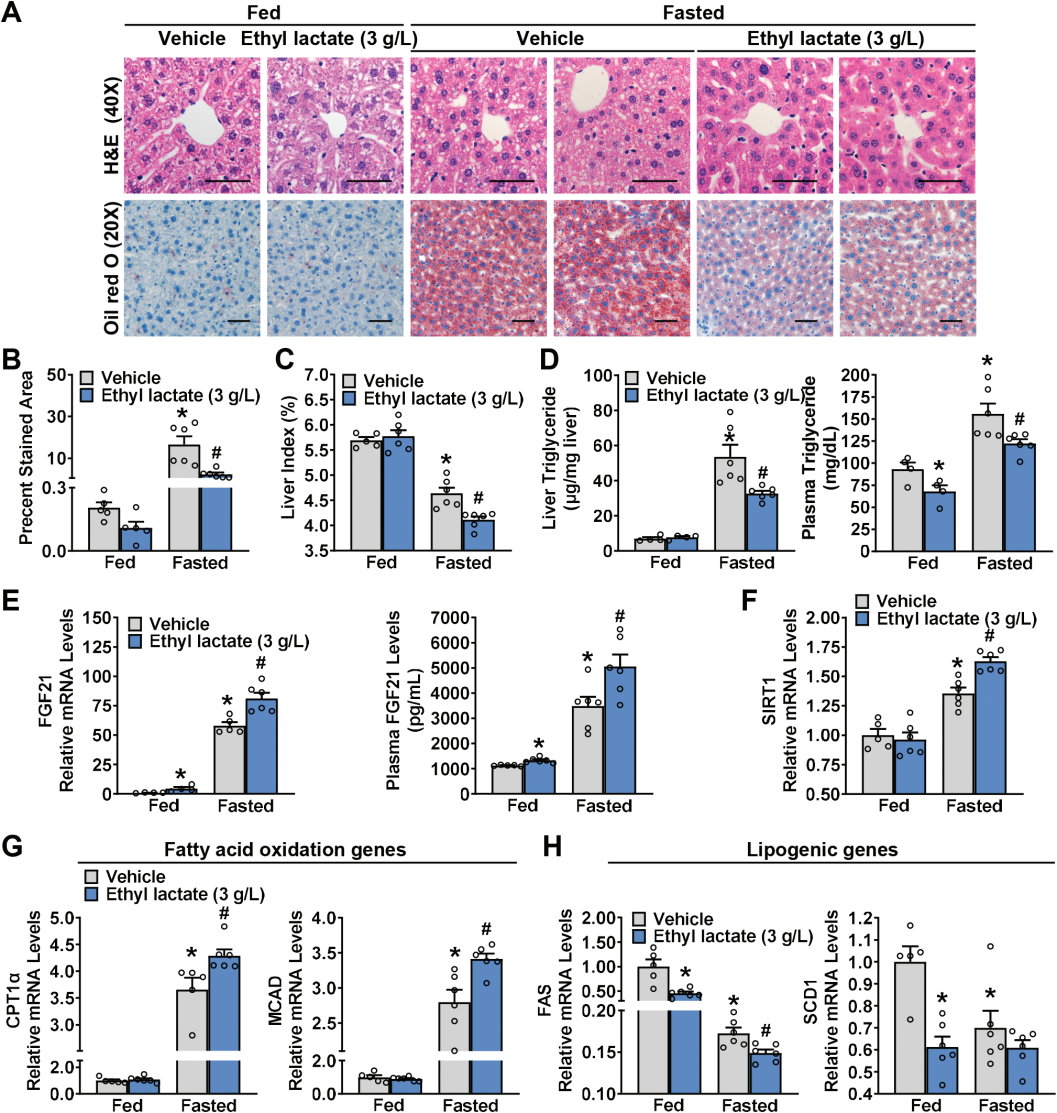
Ethyl lactate stimulates the production and secretion of hepatic FGF21 and protects against fasting‐induced hepatic steatosis in mice. WT mice were fed with normal chow diet and water containing ethyl lactate (3 g L^−1^ in water) for 1 month, followed by intraperitoneal injection of ethyl lactate (30 mg kg^−1^) or vehicle daily in the last week, and mice were fasted for 24 h. Representative H&E, Oil red O staining of liver sections A) and the quantification Oil red O‐stained area B) are shown (Scale bar: 50 µm). C) Liver index of mice. D) Hepatic TG levels and plasma TG levels in mice. E,F) Ethyl lactate continuedly increased hepatic and plasma FGF21 levels (E) as well as SIRT1 levels (F) in the liver induced by prolonged fasting. G) Hepatic expression of key genes involving fatty acid oxidation (CPT1α, MCAD) induced by fasting were further increased by ethyl lactate. H) Hepatic expression levels of key genes of lipogenesis (FAS, SCD1) inhibited by fasting were further decreased by ethyl lactate treatment. n = 4–6. ^*^ *p* < 0.05 versus fed mice treated with vehicle, ^#^ *p* < 0.05 versus fasted mice treated with vehicle.

FGF21 is a hormone predominantly secreted by the liver in response to metabolic stresses including alcohol and starvation.^[^ ^21^, ^22^ ^]^ To directly assess whether ethyl lactate is responsible for hepatic FGF21 induction after prolonged starvation, mice were administrated with drinking water containing ethyl lactate (3 g L^−1^) ad libitum for 1 month and intraperitoneal injected with ethyl lactate (30 mg kg^−1^) daily in the last week, then subjected to a 24 h fasting challenge. Surprisingly, administration of ethyl lactate significantly reduced hepatic steatosis and plasma triglyceride levels in fasted mice, without significantly altering body weight, food intake, and water intake. (**Figure** **5**A–D; Figure S4, Supporting Information). Consistently, the expression levels of FGF21 and SIRT1 in the livers of mice during the fasting state were notably increased, which were further significantly induced by ethyl lactate (Figure 5E,F).

Meanwhile, FGF21 is known as the effective regulator of hepatic fatty acid oxidation and biosynthesis.^[^ ^28^ ^]^ To determine the functional consequences of enhanced FGF21 in ethyl lactate‐treated mice, the expression levels of genes related to fatty acid oxidation and lipogenesis in the liver of mice under fasted conditions were assessed. Interestingly, mRNA levels of CPT1α and MCAD were significantly increased and further induced by ethyl lactate (Figure 5G). On the contrary, the down‐regulated FAS and SCD1 under the fasting state were further inhibited by ethyl lactate (Figure 5H). Collectively, ethyl lactate inhibits hepatic lipogenesis and improves fasting‐induced lipid accumulation in livers via FGF21.

### 2.6 Ethyl Lactate's Effects on Alcohol‐Induced Hepatic Steatosis, Inflammation, and Liver Injury in Mice are Disrupted by Pharmacologic Inhibition of SIRT1

**Figure 6:**
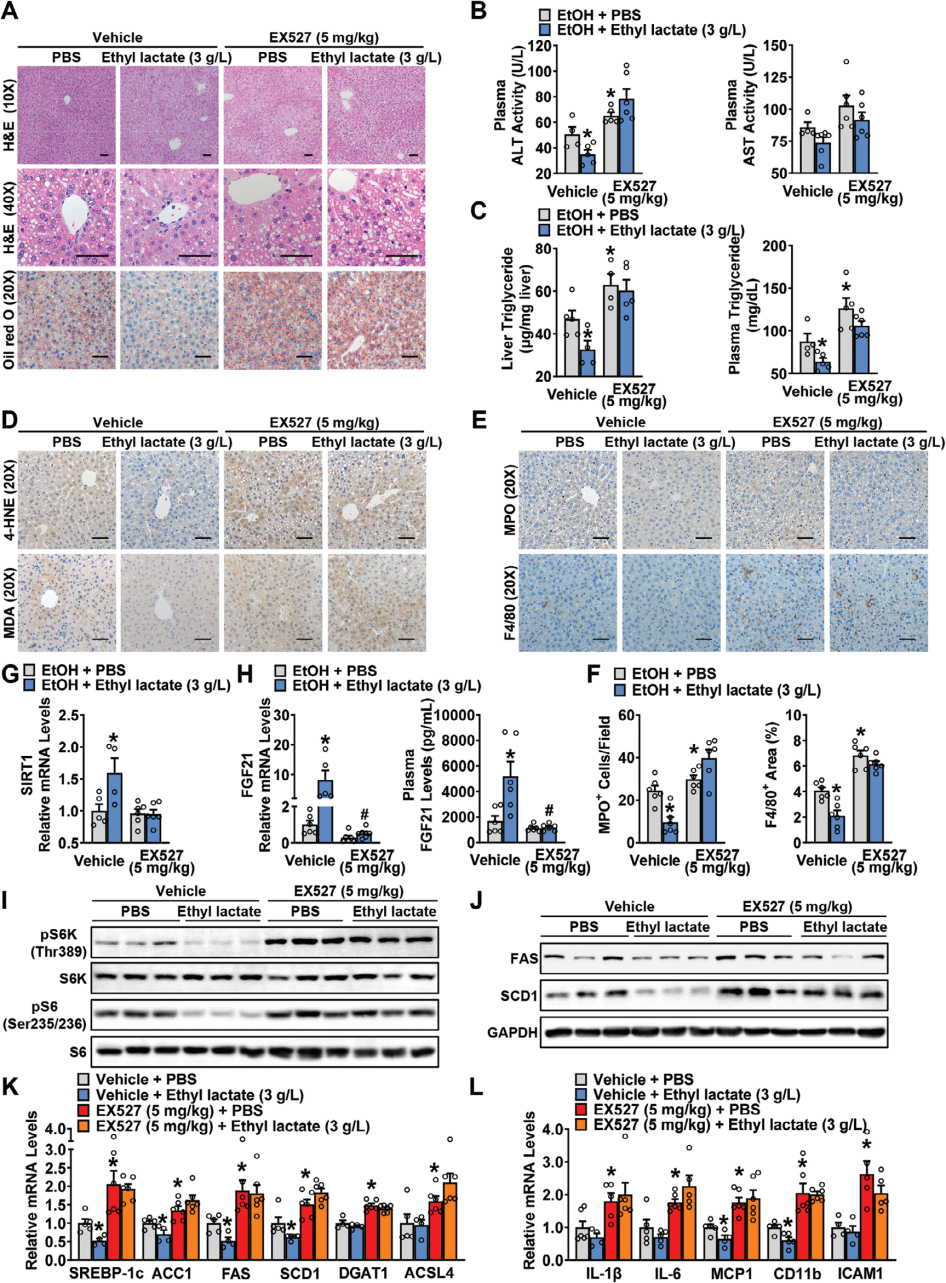
Pharmacologic inhibition of SIRT1 disrupts the effects of ethyl lactate on alcohol‐induced hepatic steatosis, inflammation, and liver injury in mice. WT mice were subjected to chronic‐plus‐binge ethanol feeding with/without ethyl lactate (3 g L^−1^) treatment, followed by intraperitoneal injection of EX527 (5 mg kg^−1^) or vehicle daily in the last 10 days. A) Representative H&E and Oil red O staining in the liver sections of mice (Scale bars: 50 µm). B) Plasma ALT and AST activity levels. C) Hepatic TG levels and plasma levels of TG. D,E) Representative IHC staining with antibody against 4‐HNE, MDA (D), MPO, and F4/80 (E). Scale bars: 50 µm. F) Quantification of number of MPO^+^ cells and percentage of F4/80^+^ area. G) Transcriptional levels of SIRT1 in the liver of mice. H) Hepatic expression of FGF21 and plasma FGF21 levels of mice. I,J) Immunoblot analysis of mTORC1 activities (I) and lipogenesis (J) were performed. n = 3/group. K,L) The transcriptional levels of lipogenic genes (K) and inflammatory genes (L) in the liver of mice. n = 4‐6. ^*^ *p* < 0.05 versus vehicle and PBS, ^#^ *p* < 0.05 versus vehicle and ethyl lactate.

To further confirm the necessity of SIRT1 in mediating the attenuation of ASH through the induction of FGF21, during chronic ethanol feeding, a specific SIRT1 inhibitor EX527 (5 mg kg^−1^) was administrated daily through intraperitoneal injection. Strikingly, EX527 blocked the beneficial effects of ethyl lactate on alcohol‐induced hepatic steatosis, injury, oxidative stress, and inflammation (**Figure** **6**A–F; Figure S5, Supporting Information). Furthermore, the inhibition of SIRT1 disrupted both hepatic FGF21 expression and plasma FGF21 levels induced by ethyl lactate (Figure 6G,H). Consistent with these findings, the ethyl lactate‐caused inhibition of phosphorylation of mTORC1 substrates S6K and S6, as well as lipogenic proteins, was abolished in the livers of mice treated with EX527(Figure 6I,J). Likewise, the inhibitory effects of ethyl lactate on alcohol‐induced lipogenic and inflammatory genes were eliminated by EX527 administration (Figure 6K,L). These results indicate that SIRT1 is required for ethyl lactate‐induced FGF21 production and hepatic improvement.

### 2.7 Ethyl Lactate's Effects on the Reduction of mTORC1 Activity and Lipogenesis and Improvement of the Development of Alcoholic Fatty Liver and Liver Injury in Mice are Abolished by Hepatocyte‐Specific Deletion of FGF21

**Figure 7:**
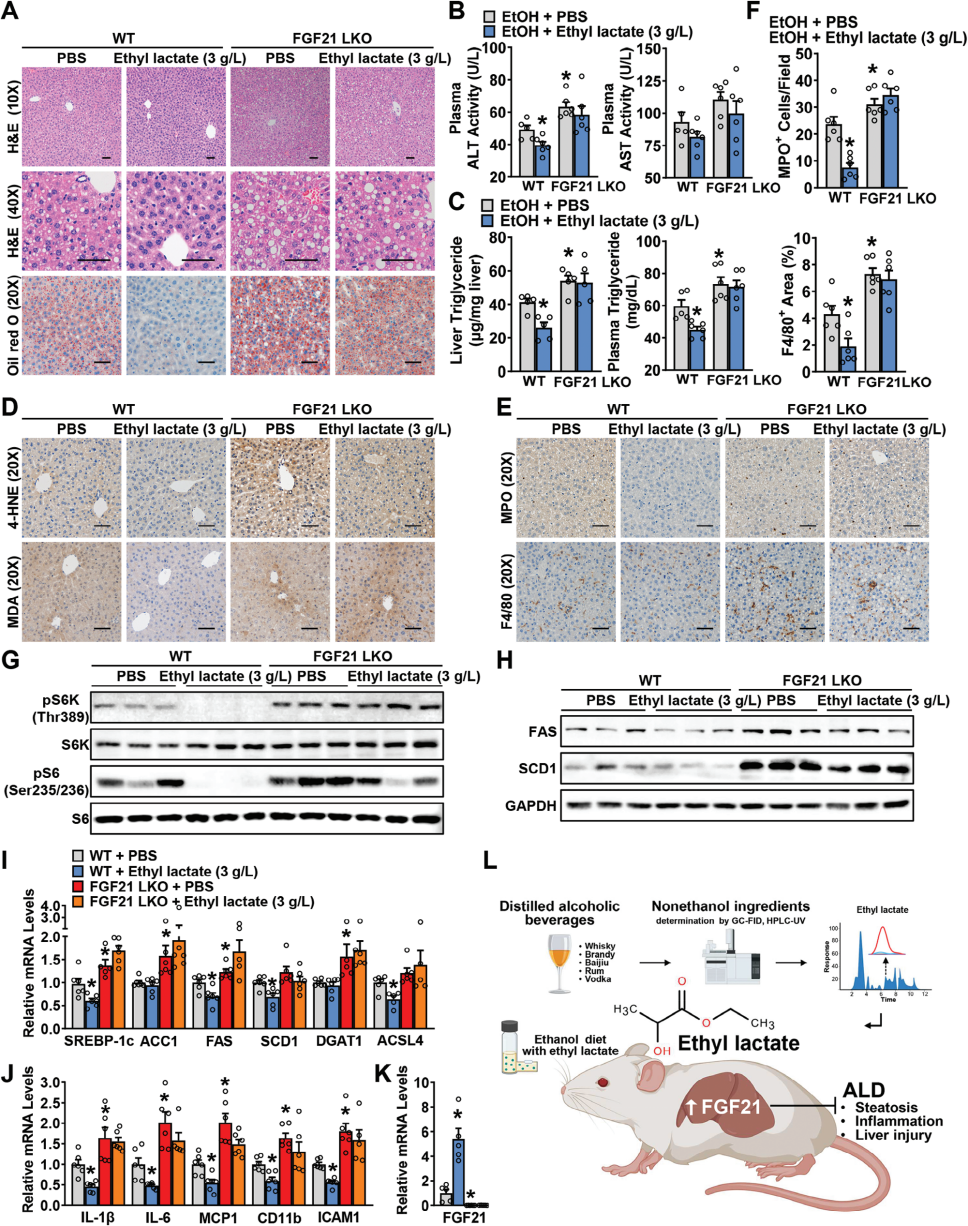
Hepatocyte‐specific deletion of FGF21 abolishes the effects of ethyl lactate on reducing mTORC1 activity and lipogenesis and exacerbates the development of alcoholic fatty liver and liver injury in mice. FGF21 LKO mice and their wild‐type littermates were subjected to chronic‐plus‐binge ethanol feeding with/without ethyl lactate (3 g L^−1^) treatment. A) Representative H&E and Oil red O staining in the liver sections of mice (Scale bars: 50 µm). B) Plasma ALT and AST activity levels. C) Hepatic TG levels and plasma TG levels. D,E) Representative IHC staining with antibody against 4‐HNE, MDA (D), MPO, and F4/80 (E). Scale bars: 50 µm. F) Quantification of number of MPO^+^ cells and percentage of F4/80^+^ area. G,H) Immunoblot analysis of mTORC1 activities (G) and lipogenesis (H) were performed. n = 3/group. I,J) The transcriptional levels of lipogenic genes (I) and inflammatory genes (J) in the liver of mice. K) Expression levels of FGF21 in the liver of mice. n = 5–6. ^*^ *p* < 0.05 versus WT mice treated with PBS. L) The proposed model for nonethanol ingredient ethyl lactate‐mediated hepatic FGF21 activation in inhibiting the pathogenesis of ALD in mice. In the progression of ALD, ethyl lactate, which is enriched in spirits, ameliorates alcohol‐induced hepatic steatosis, inflammation, and acute‐on‐chronic liver injury. The production of hepatic FGF21 is stimulated by ethyl lactate and mediates the inhibitory effects of ethyl lactate on lipogenesis and ALD progression. Targeting ethyl lactate may represent a potential strategy for the treatment of ALD via inhibiting lipogenesis.

To further establish an indispensable role of FGF21 in the regulatory effects of ethyl lactate on the development of ALD, liver‐specific FGF21 knockout (FGF21 LKO) mice were generated (**Figure** **7**K). Compared with WT littermates, FGF21 LKO mice developed more severe hepatic steatosis, injury, dyslipidemia, oxidative stress, and inflammation with ethanol feeding.^[^ ^29^ ^]^ Hepatic FGF21 deficiency abolished the effects of ethyl lactate on improving ASH (Figure 7A–F; Figure S6, Supporting Information). Furthermore, the inhibitory effects of ethyl lactate on mTORC1 hyperactivation and lipogenesis were diminished by hepatic FGF21 deficiency (Figure 7G,H). Similarly, FGF21 LKO abrogated the ethyl lactate's beneficial effects on normalizing expressions of key lipogenic and inflammatory genes in livers (Figure 7I,J). These results suggest a critical role in hepatic FGF21 in mediating ethyl lactate‐induced alleviation of ALD in mice fed with a chronic‐plus‐binge ethanol diet.

## 3 Discussion

The present study reveals for the first time a novel small molecule of ethyl lactate, a representative nonethanol ingredient in distilled alcoholic beverages, with the potential to against chronic‐plus‐binge ethanol feeding‐induced hepatic steatosis, inflammation, and injury in mice. Mechanistically, ethyl lactate stimulates hepatocellular FGF21 expression and production dose‐dependently, exerting the inhibitory effects of FGF21 on alcohol‐induced aberrant activation of mTORC1 to suppress hepatic lipogenesis. Thus, ethyl lactate attenuates ALD in a hepatic FGF21‐dependent manner in mice (Figure 7L).

### 3.1 Ethyl Lactate Represents a Nonethanol Ingredient of Distilled Liquor Containing the Inhibitory Effect on Alcohol‐Induced Lipogenesis

Aberrant upregulation of hepatic lipogenesis is associated with a wide variety of pathologies, so inhibiting core enzymes of lipogenesis represents an attractive therapeutic strategy.^[^ ^11^ ^]^ Here, we identified a novel small molecule of ethyl lactate as an inhibitor of hepatic lipogenesis, showing inhibitory effects on alcohol‐induced lipogenesis. SREBP‐1c‐dependent lipogenesis is increased in mice and humans with ALD,^[^ ^9^ ^]^ and ethyl lactate significantly lowers mRNA and protein levels of key lipogenic enzymes regulated by SREBP‐1c, such as FAS and SCD1, in livers of chronic‐plus‐binge ethanol‐fed mice. Moreover, ethyl lactate also inhibits hepatic lipogenesis under fasted physiological conditions, suggesting that ethyl lactate could be an effective inhibitor of lipogenesis for the treatment of metabolic disorders, such as metabolic dysfunction‐associated steatotic liver disease (MASLD).

Fermented foods and their components are thought to have a beneficial effect on various aspects of human health, especially gastrointestinal health.^[^ ^30^ ^]^ However, the effects of nonethanol ingredients which represent the fermented metabolites of alcohol fermentation on human health are poorly understood. Till now, over one thousand chemicals have been identified in distilled spirits,^[^ ^19^ ^]^ and it has been reported that some molecules have bioactivity in regulating pathophysiological processes, such as butyric acid in hepatosteatosis^[^ ^31^ ^]^ and lactate in immunopathology.^[^ ^32^ ^]^ Notably, ethyl lactate is a common component in a range of fermented foods beyond alcoholic beverages, such as sausage, vinegar, and bread,^[^ ^33^, ^34^, ^35^, ^36^ ^]^ and is also utilized as a food flavoring agent that is approved by the Joint FAO/WHO Expert Committee on Food Additives. Thus, the biological function of foodborne chemical components,^[^ ^30^ ^]^ such as nonethanol ingredients of distilled spirits, are worthy of more investigation.

### 3.2 Ethyl Lactate Protects Against ALD Pathogenesis by Inducing the Hepatokine FGF21

One of the major findings of this study is the demonstration of the nonnegligible roles of nonethanol ingredients of distilled spirits in regulating the progression of ALD, which may provide new insights into the complex pathogenesis of ALD. Here, we identified the beneficial effects of ethyl lactate on alcohol‐induced hepatic steatosis, inflammation, and injury in a dose‐dependent manner. RNA‐sequencing analysis revealed that ethyl lactate significantly normalized the transcriptional profiles in the livers of ethanol‐fed mice, especially in lipid metabolism and inflammation pathways. Coincidentally, ethyl pyruvate, a structural analog of ethyl lactate, has shown remarkable pharmacological effects of anti‐inflammation and anti‐injury in a wide variety of preclinical models of critical illnesses, such as severe sepsis.^[^ ^37^, ^38^, ^39^ ^]^ These findings provide good support for further translational and clinical development of ethyl lactate to treat human ALD.

FGF21 is a stress‐inducible hormone that exhibited important roles in maintaining metabolic balance through a heterodimeric receptor complex comprising FGF receptor 1 (FGFR1) and βklotho.^[^ ^23^ ^]^ In clinical trials, FGF21 analogs show therapeutic efficacy in glycaemic control, weight loss, alleviating dyslipidaemia,^[^ ^40^, ^41^ ^]^ improving metabolic dysfunction‐associated steatohepatitis (MASH) and liver fibrosis.^[^ ^42^, ^43^ ^]^ This present study shows that ethyl lactate is an effective FGF21 activator both under physiological and pathological conditions, as evidenced by the hepatic mRNA levels and plasma levels of FGF21. Furthermore, the loss‐of‐function of FGF21 illustrates the necessity of FGF21 for the beneficial effects of ethyl lactate on alcoholic steatosis, inflammation, and injury. Given that FGF21 and its analogs have been extensively shown to be beneficial for treating obesity, type 2 diabetes, and MASLD in a number of animal models and humans, ethyl lactate, a dietary chemical component, may have therapeutic potential for treating alcoholic liver diseases and other metabolic disorders. Together, the current study characterizes ethyl lactate as a novel activator of FGF21, which may represent a cellular mechanism in regulating ALD pathogenesis.

### 3.3 Ethyl Lactate Downregulates Alcohol‐Induced Hepatic Lipogenesis Through FGF21

Alcohol‐induced lipogenesis, which leads to the biosynthesis and accumulation of new fatty acids, promotes the progression of ALD.^[^ ^9^ ^]^ Chronic and excessive alcohol consumption causes sustained mTORC1 activation, thus increasing the transcriptional activity of SREBP‐1c.^[^ ^9^, ^29^ ^]^ Here, in vivo and in vitro data demonstrate that ethyl lactate suppresses alcohol‐induced activation of mTORC1 and downregulates hepatic lipogenesis. Consistently, ethyl lactate treatment significantly alleviates chronic alcohol feeding‐induced hepatic steatosis in mice and ethanol expose‐induced lipid accumulation in mouse primary hepatocytes. Our previous studies indicated that FGF21 down‐regulated mTORC1 in a tuberous sclerosis complex (TSC)‐dependent manner.^[^ ^25^ ^]^ However, this inhibitory action of FGF21 on mTORC1 in ALD is little known. Hepatic FGF21 deficiency causes hyperactivation of mTORC1, resulting in a hyperactive lipogenic program and exacerbated alcoholic hepatosteatosis, inflammation, and injury. The suppressive effects of ethyl lactate on mTORC1 are ablated in FGF21 LKO mice. Therefore, ethyl lactate downregulates alcohol‐induced lipogenesis by stimulating FGF21. Interestingly, cholesterol 7α‐hydroxylase (Cyp7a1), the critical rate‐limiting and regulatory enzyme in bile acid synthesis,^[^ ^44^ ^]^ was also upregulated by ethyl lactate treatment. Whether Cyp7a1‐regulated bile acid homeostasis plays a role in the beneficial effects of ethyl lactate‐FGF21 signaling on ALD progression requires future studies.

In response to excessive alcohol intake, FGF21 is robustly activated to play a compensatory role in protecting against metabolic stress, but the mechanism underlying endocrine regulation of the alcohol response of FGF21 is obscure. It has been reported that the transcription of FGF21 is controlled by nutritional status, such as fasting and refeeding regimens.^[^ ^45^ ^]^ Our previous studies revealed that SIRT1‐mediated activation of FGF21 prevents liver steatosis caused by fasting.^[^ ^26^ ^]^ Here, the inhibition of SIRT1 by EX527 prominently abolishes the expression and production of hepatic FGF21, leading to a more severe ALD phenotypes. The inductions of FGF21 by ethyl lactate are also ablated, suggesting that ethyl lactate stimulates the expression and secretion of FGF21 through hepatic SIRT1. Ethyl lactate treatment increased both mRNA and protein levels of SIRT1 in the hepatocyte and mouse liver, suggesting that ethyl lactate is likely to regulate SIRT1 at the transcriptional level. The transcriptional mechanism for the effects of ethyl lactate on SIRT1 requires future investigations.

In summary, this study provides a novel insight into understanding the pathogenesis of ALD and reveals that ethyl lactate with in vivo bioactivity functions as an efficient inhibitor of lipogenesis to ameliorate alcoholic steatosis, inflammation, and acute‐on‐chronic liver injury in a FGF21‐dependent manner. Approaches for the modulation of the foodborne molecule ethyl lactate may provide a unique therapeutic pathway for the treatment of ALD.

## 4 Experimental Section

### Animal Models

Liver‐specific FGF21 knockout mice (FGF21 LKO) were generated by crossing the FGF21‐floxed mice with albumin‐Cre mice as previously described.^[^ ^46^ ^]^ Female and male mice (age of 10–14 weeks) were placed on a chronic–binge ethanol diet (Gao‐Binge model) as previously described.^[^ ^18^ ^]^ Mice were fed with Lieber–DeCarli diet and the caloric intake from ethanol was 0% on days 1–5 and 36% from day 6 until the end of the study period. On day 16, mice were gavaged with a single dose of ethanol (5 g kg^−1^ body weight) in the early morning and sacrificed 9 h later. Pair‐fed control mice received a diet with an isocaloric substitution of dextrose. For the nonethanol ingredient treatment, each compound was first added to 52% ethanol and then mixed into the ethanol diet (5% v/v) during the chronic ethanol feeding phase, or it was directly used for ethanol gavage. For SIRT1 inhibitor treatment, mice were fed a chronic‐binge ethanol diet and concurrently administered 5 mg kg^−1^ SIRT1 inhibitor EX527 (cat. S1541, Selleck) or vehicle (5% DMSO, 30% polyethylene glycol, and 65% saline) via daily intraperitoneal injections over the final 10 days of the study. For feeding and fasting mouse experiments, 8‐week male C57BL/6 mice were placed on a normal chow diet and drinking water with or without 3 g L^−1^ ethyl lactate for 1 month, followed by 30 mg kg^−1^ ethyl lactate or vehicle injection for 1 week before 24 h fasting. The fed group was placed on a normal chow diet, while the fasted group was fasted for 24 h, and sacrificed at the end of the dark cycle.^[^ ^26^ ^]^ Female and male C57BL/6 mice at 8 weeks of age were purchased from Shanghai Laboratory Animal Co. (Shanghai, China). All mice were housed under a 12:12 h light/dark cycle at a controlled temperature. All animal experimental protocols were approved by the Institutional Animal Care and Use Committee at the Shanghai Institute of Nutrition and Health, Chinese Academy of Sciences (SINH‐2023‐LY‐1).

### Quantitative Analysis of Nonethanol Ingredients in Distilled Liquors

An Agilent 7890 gas chromatograph equipped with an FID was used for volatile compounds analysis, as described previously.^[^ ^17^ ^]^ 1 µL sample was directly injected into the GC‐FID after filtration. The GC injector temperature was maintained at 250 °C for HP‐INNOWAX column (30 m × 0.32 mm × 0.25 µm, Agilent Technologies, USA), the split ratio was 10:1, and the oven temperature was initially held at 35 °C for 1 min, then raised to 50 °C at 3 °C min^−1^, increased at 5 °C min^−1^ to 230 °C and finally held for 13 min. Nitrogen was used as the carrier gas at a fixed flow rate of 0.8 mL min^−1^. For quantitative analysis of nonvolatile compounds, The Agilent 1200 series HPLC equipped with a UV detector was used. The nonvolatile compounds were separated using an HPX‐87H column (300 mm × 7.8 mm × 9 µm) at 60 °C and detected at 210 nm wavelength. The mobile phase was the dilute sulfuric acid solution (5 mM) with a flow rate at of 0.6 mL min^−1^ and the elution time was 30 min. The injection volume was 20 µL. All analyses were repeated in triplicate.

### Cell Treatment

Primary mouse hepatocytes were isolated using the method described previously^[^ ^9^ ^]^ and cultured in DMEM (Gibco, 1 g L^−1^ D‐glucose, MA, USA). The alpha mouse liver 12 (AML12) hepatocytes were purchased from the National Collection of Authenticated Cell Cultures were cultured in DMEM (Gibco, 4.5 g L^−1^ D‐glucose, MA, USA). The cells were grown and maintained in a humidified chamber with 5% CO_2_ at 37 °C. For ethanol treatment, cells were seeded in 6‐well plates and cultured overnight to reach ≈80% confluence, and then incubated for 48 h with 100 mM ethanol and ethyl lactate (1, 10 mM) in serum‐free medium. And during ethanol and ethyl lactate incubation, the medium containing ethanol and ethyl lactate needed to be replaced in 24 h.

### Statistical Analysis

Data were expressed as mean ± SEM. Statistical significance was evaluated using the unpaired two‐tailed Student's *t*‐test for two groups. For more than two groups, data were analyzed by one‐way ANOVA or two‐way ANOVA. Differences were considered significant at *p* value < 0.05.

## Conflict of Interest

The authors declare no conflict of interest.

## Author Contributions

Y.J., D.X., A.C., Y.Z., C.Z., and Y.L. contributed to the experiment design. Y.J., S.W., S.S., Y.L., W.S., D.D., Z.Z., H.Y., T.Z., Q.Y., J.Z., X.F., L.L., and A.C. contributed to the acquisition and analysis of data. Y.S. and Y.Z. edited the manuscript with important intellectual content. A.C., Q.W., C.Z., and Y.L. obtained the funding. Y.J., S.W., A.C., Q.W., C.Z., and Y.L. wrote the manuscript.

## Supporting information



Supporting Information

## Data Availability

The data that support the findings of this study are available from the corresponding author upon reasonable request.
