# Supplementary material for: Ethyl Lactate Ameliorates Hepatic Steatosis and Acute‐on‐Chronic Liver Injury in Alcohol‐Associated Liver Disease by Inducing Fibroblast Growth Factor 21
Source: Adv Sci (Weinh). 2024 Dec 11;12(5):2409516. doi: 10.1002/advs.202409516 (PMC11792039; doi:10.1002/advs.202409516)
Supplement: Supplementary file 1 — Supporting Information [file ADVS-12-2409516-s001.docx]

**Ethyl lactate ameliorates hepatic steatosis and acute-on-chronic liver injury in alcohol-associated liver disease by inducing fibroblast growth factor 21**

Yang Jiang^1,2,6^, Shuang Wei^2,6^, Shiming Shen^1^, Yuxiao Liu^2^, Weitong Su^2^, Dong Ding^2^, Zengpeng Zheng^2^, Haokai Yu^1,2^, Tingting Zhang^3^, Qiuli Yang^3^, Jiuxiang Zhao^3^, Yi Shen^4^, Xia Fang^1,5^, Liangcai Lin^1^, Dongguang Xiao^1^, Aoyuan Cui^2^*, Qin Wan^5^*, Yadong Zhang^4^*, Yu Li^2,3^*, Cuiying Zhang^1^*

^1^State Key Laboratory of Food Nutrition and Safety, College of Biotechnology, Tianjin University of Science and Technology, Tianjin 300457, China; ^2^CAS Key Laboratory of Nutrition, Metabolism and Food Safety, Shanghai Institute of Nutrition and Health, University of Chinese Academy of Sciences, Chinese Academy of Sciences, Shanghai 200031, China; ^3^CAS Engineering Laboratory for Nutrition, Shanghai Institute of Nutrition and Health, University of Chinese Academy of Sciences, Chinese Academy of Sciences, Shanghai 200031, China; ^4^Sichuan Langjiu Co., Ltd, Gulin, Sichuan 646523, China; ^5^Department of Endocrinology and Metabolism, Metabolic Vascular Disease Key Laboratory of Sichuan Province, The Affiliated Hospital of Southwest Medical University, Luzhou, Sichuan 646000, China; ^6^These authors contribute equally to this work;

**
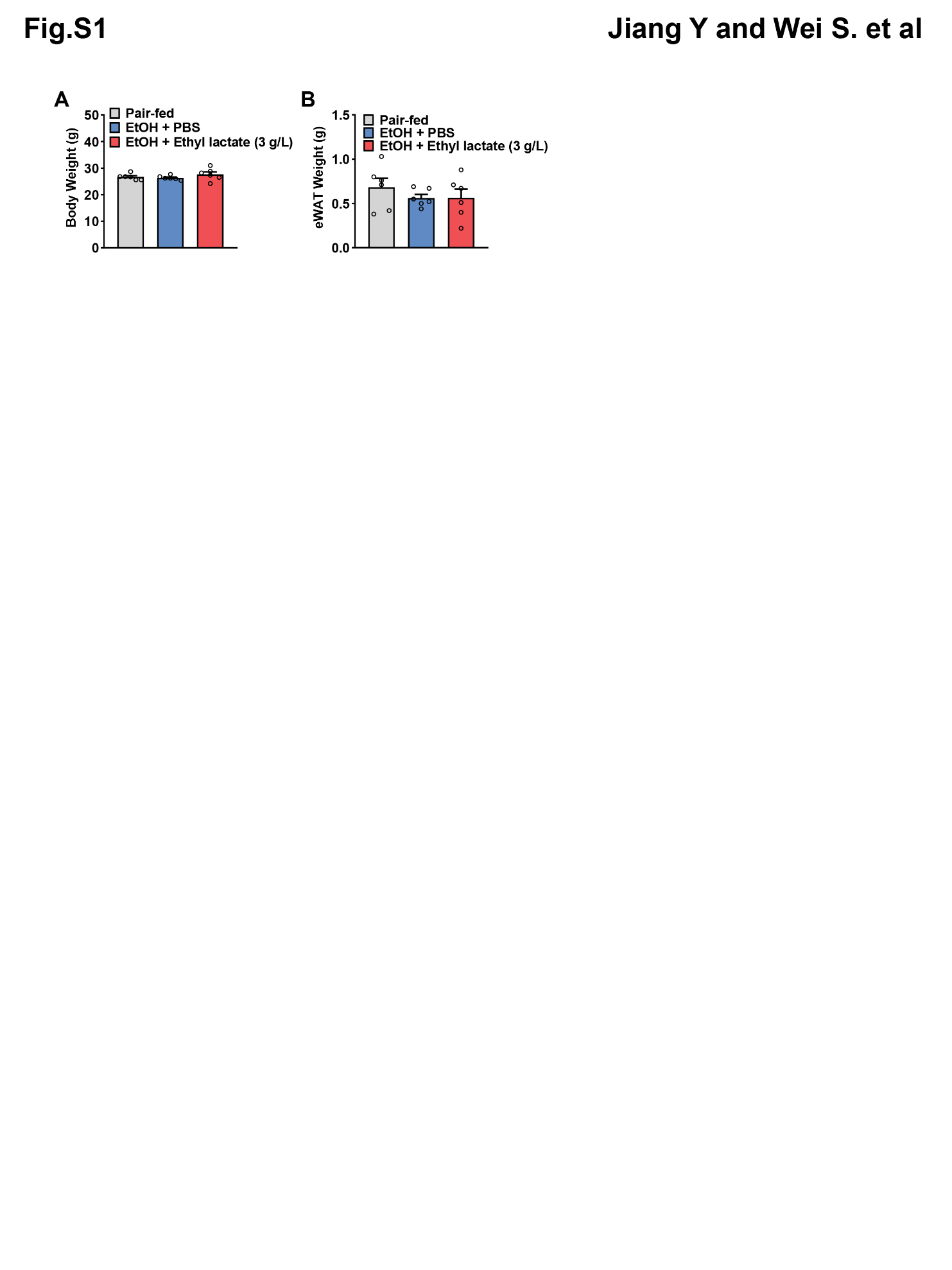

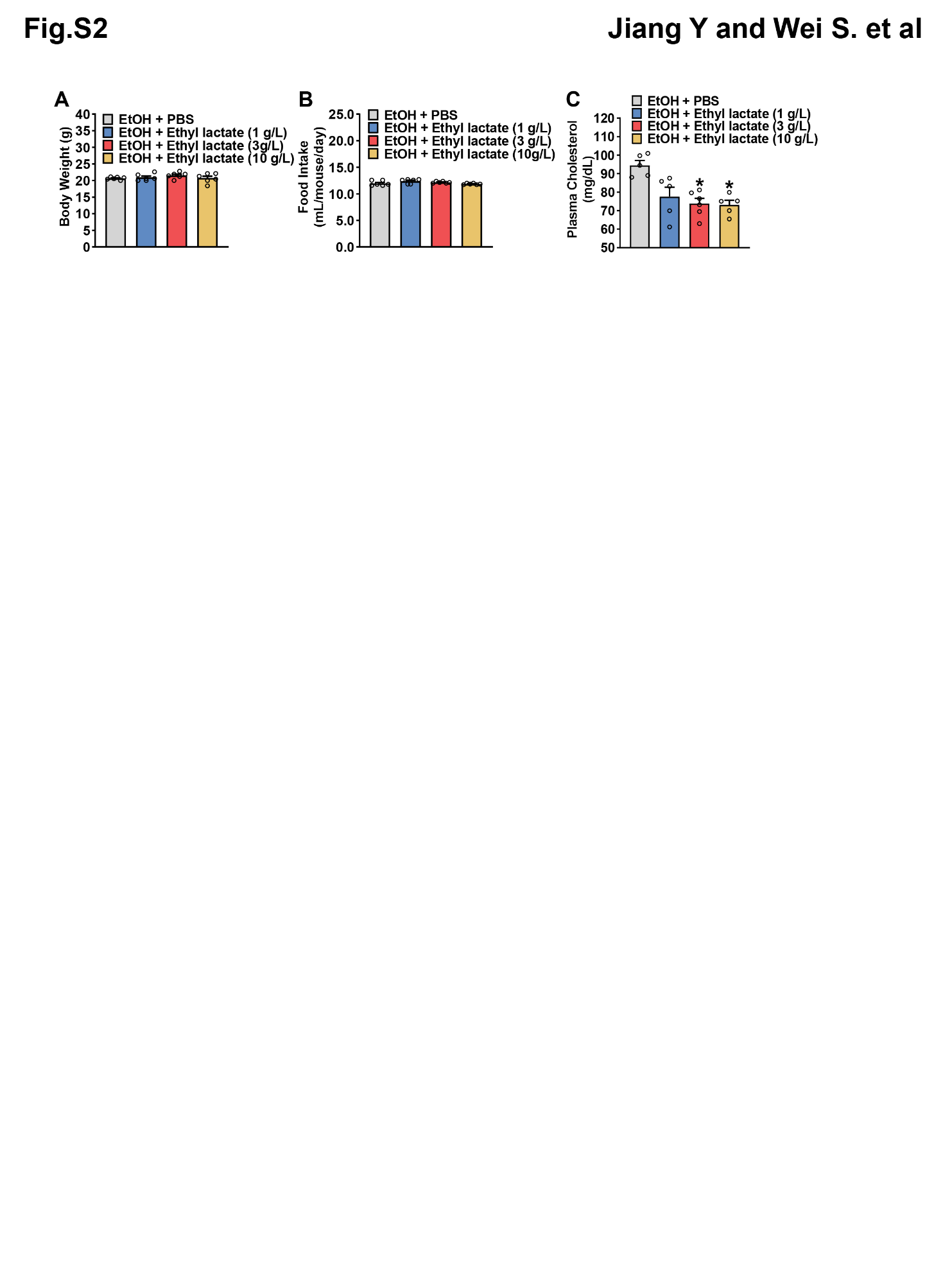

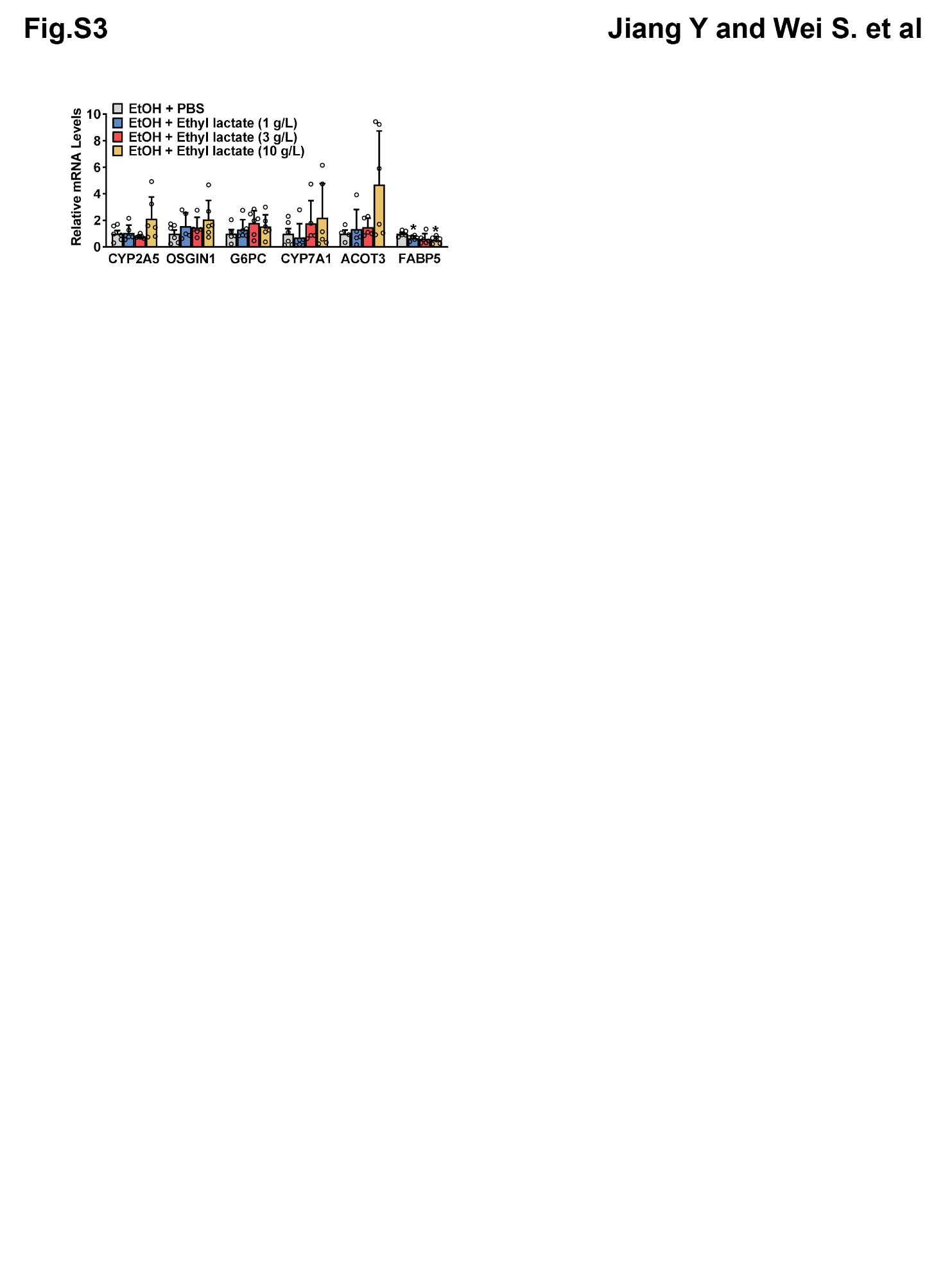

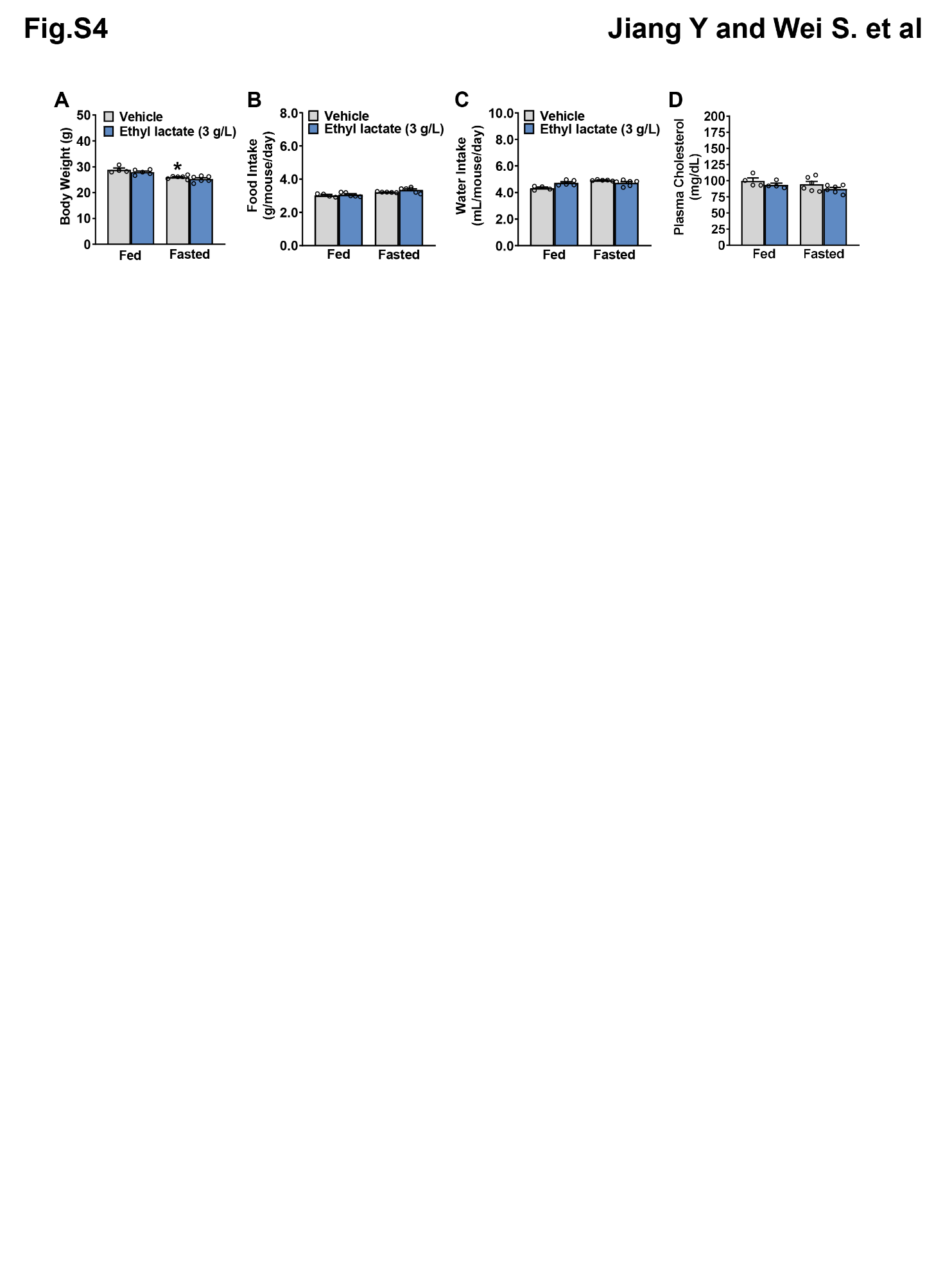

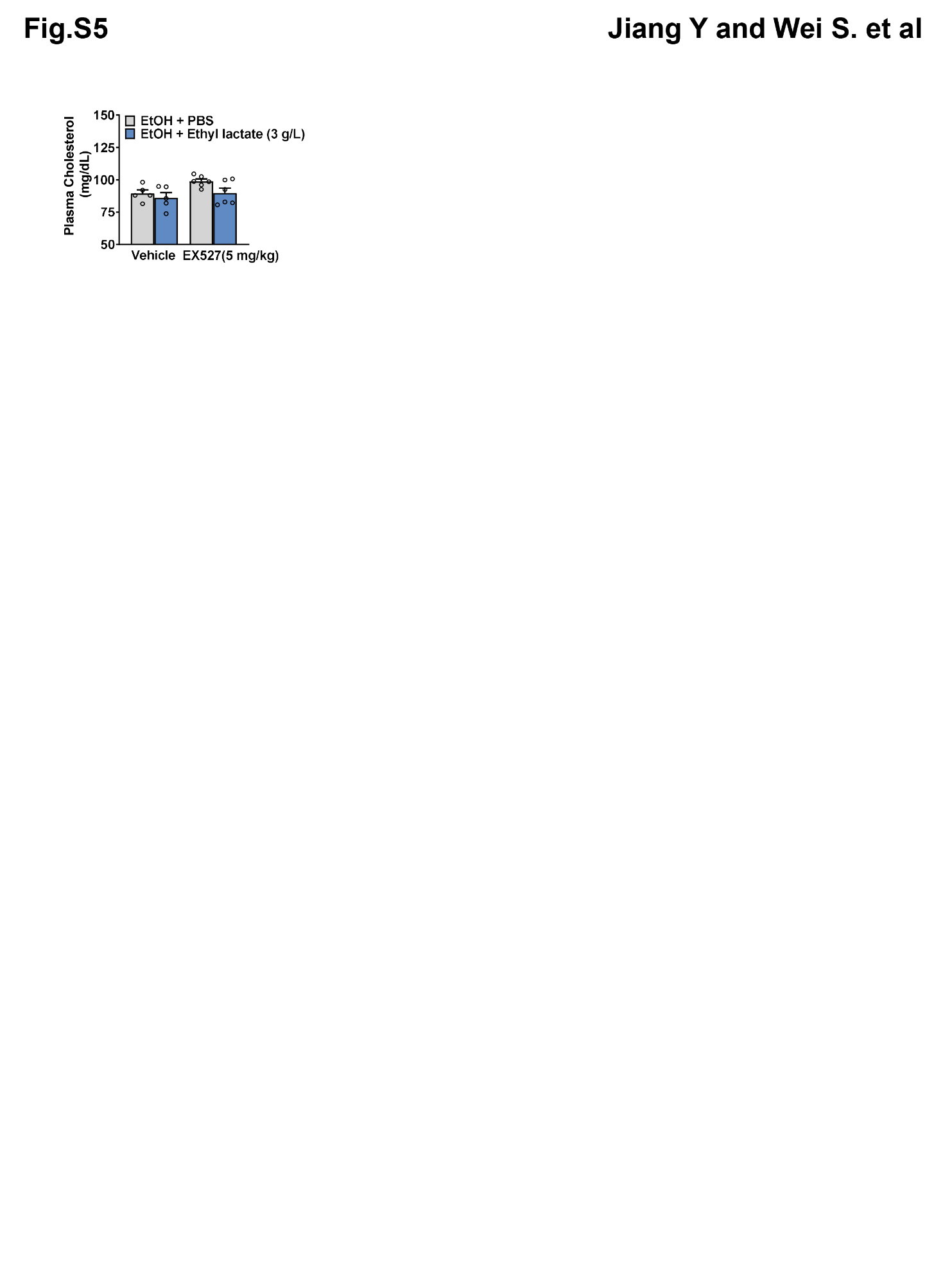

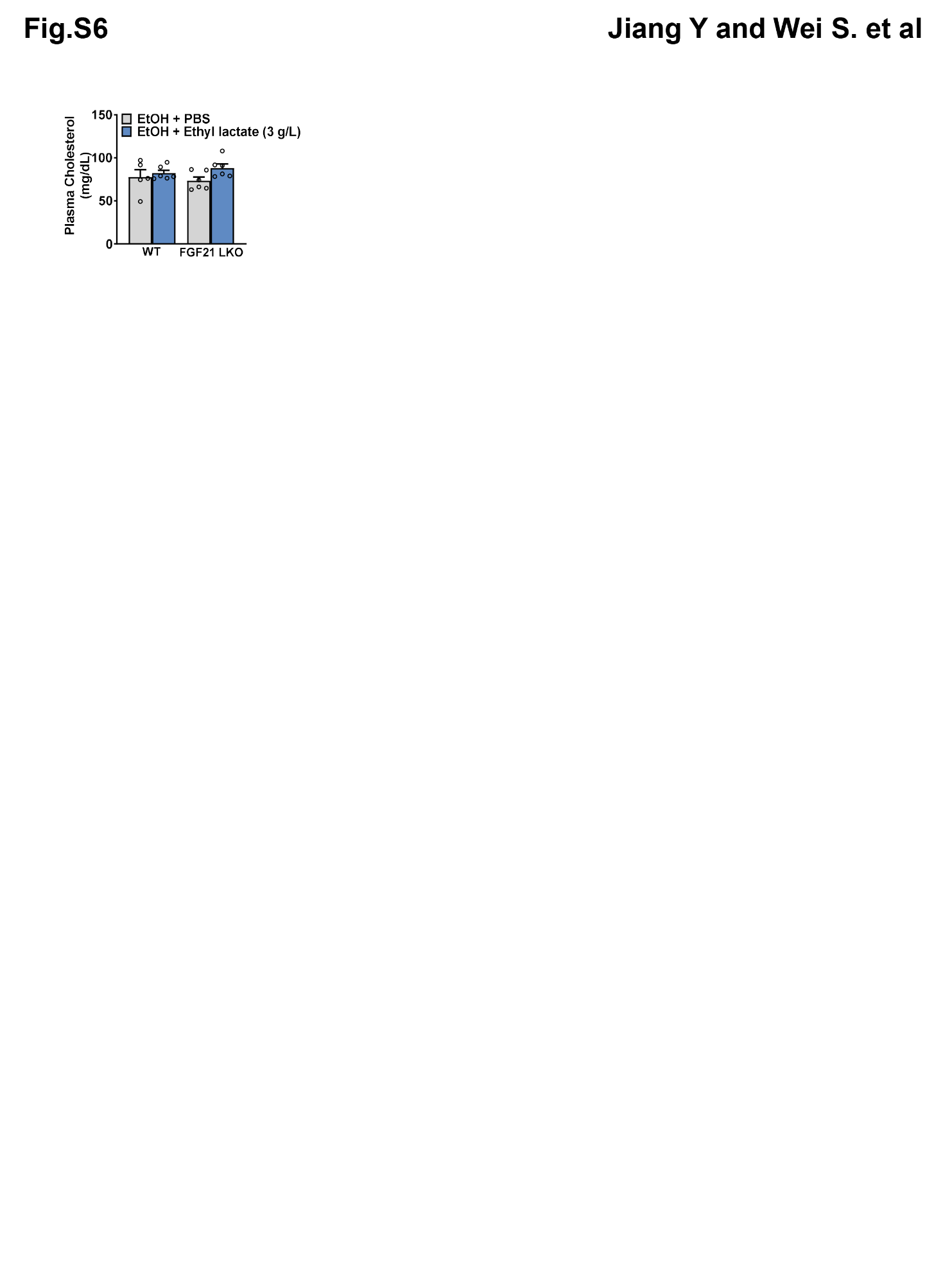
Supplemental Figure Legends**

**Fig.S1.** **Ethyl lactate exhibited moderate effects on the body weight and** **epididymal white adipose tissue weight in chronic-plus-binge ethanol fed mice.** WT mice were fed a 5% (v/v) ethanol diet for 10 days, followed by gavage of ethanol (5 g/kg), and were euthanized 9 hours later (NIAAA model). Ethyl lactate (3 g/L) was added in 52% (v/v) ethanol and then mixed in chronic-plus-binge ethanol diet. (A) Body weight. (B) Epididymal white adipose tissue (eWAT) weight. n=6.

**Fig.S2. The body weight and food intake of mice feeding chronic-plus-binge ethanol.** WT mice were subjected to chronic-plus-binge ethanol feeding, and a range dose of ethyl lactate (1, 3, 10 g/L) was added in 52% (v/v) ethanol and then mixed in chronic-plus-binge ethanol diet. (A) Body weight. (B) Food intake. (C) Plasma cholesterol levels. n=5-6. *p<0.05, vs. pair-fed.

**Fig.S3. Verification of differentially expressed genes regulated by ethyl lactate.** WT mice were subjected to chronic-plus-binge ethanol feeding, and a range dose of ethyl lactate (1, 3, 10 g/L) was added in 52% (v/v) ethanol and then mixed in chronic-plus-binge ethanol diet. mRNA levels of CYP2A5, OSGIN1, G6PC, CYP7A1, ACOT3 and FABP5 in livers of mice were determined by real-time PCR. n=4-6. *p<0.05, vs. ethanol-fed with PBS.

**Fig.S4. The body weight, food and water intake of mice under fed or fated conditions.** WT mice were fed with normal chow diet and water containing ethyl lactate (3 g/L in water) for 1 month, followed by intraperitoneal injection of ethyl lactate (30 mg/kg) or vehicle daily in the last week, and mice were fasted for 24 h before sacrifice. n=4-6. (A) Body weight. (B) Food intake. (C) Water intake. (D) Plasma cholesterol levels. *p<0.05, vs. fed mice treated with vehicle.

**Fig.S5. Plasma cholesterol levels of mice treated with SIRT1 inhibitor.** WT mice were subjected to chronic-plus-binge ethanol feeding with/without ethyl lactate (3 g/L) treatment, followed by intraperitoneal injection of EX527 (5 mg/kg) or vehicle daily in the last 10 days. n = 4-6.

**Fig.S6. Plasma cholesterol levels of FGF21 LKO mice.** FGF21 LKO mice and their wild-type littermates were subjected to chronic-plus-binge ethanol feeding with/without ethyl lactate (3 g/L) treatment. n=4-6.

**Supplemental Experimental Procedures**

**Reagents and antibodies—**Ethanol (standard for GC, >99.8%) (cat. E111962) and ethyl lactate (standard for GC, ≥99.0%) were purchased from Aladdin (Shanghai, China). Lieber-DeCarli control liquid diet (cat. TP4030C) and Lieber-DeCarli ethanol diet (cat. TP4030D) were purchased from Trophic (Nantong, China). The Mouse FGF-21 ELISA Kits (cat. MF2100) were purchased from R&D Systems (Minneapolis, MN). Alanine and Aspartate Transaminase Determination Kits purchased from Shensuo UNF (Shanghai, China). Triglyceride kit (cat. A110-1-1) and total Cholesterol kit (cat. A111-1-1) purchased from Nanjing Jiancheng Biotechnology Institute (Nanjing, China). 4,4-difluoro-1,3,5,7,8-pentamethyl-4-bora-3a,4a-diaza-s-indacene (BODIPY 493/503) (cat. D3922) was purchased from Thermo Fisher Scientific (Waltham, MA). Selisistat (EX 527) (cat. S1541 ) was purchased from Selleck (Houston USA). SirT1 (1F3) mouse mAb (cat. 8469), Phospho-p70 S6 Kinase (Thr389) (108D2) (cat. #9234), p70 S6 Kinase (cat. #9202), Phospho-S6 Ribosomal Protein (Ser235/236) (cat. #2211), S6 Ribosomal protein (cat. #2217), Stearoyl-CoA desaturase 1 (M38) (cat. 2438) and GAPDH (cat. #5174) antibodies were purchased from Cell Signaling Technology (Massachusetts, USA). Fatty acid synthase mAb (cat. 610963) was purchased from BD Biosciences (New Jersey, USA).

**Liver Histological Analysis—**Hematoxylin & Eosin (H&E) staining of liver tissue sections was performed as described previously[^1^](#_ENREF_1)^,^ [^2^](#_ENREF_2). Liver tissues were rapidly harvested and fixed in phosphate-buffered 4% formalin (cat. G1101, Servicebio) overnight at 4°C. Fixed liver tissues were then embedded in paraffin. Paraffin sections (4 μm) were cut and mounted on glass slides. Paraffin-fixed liver sections were subjected to H&E staining. For liver oil red O staining, livers were embedded in ornithine carbamyl transferase (OCT) (Tissue-Tek, Laborimpex) and rapidly frozen on the dry ice, as described[^3^](#_ENREF_3). Hepatic steatosis was assessed by oil red O staining kit from American MasterTech (Lodi, CA) according to the manufacturer’s protocol.

**Primary mouse hepatocyte isolation and culture—**Primary mouse hepatocytes were isolated using a method described previously[^4-6^](#_ENREF_4). Briefly, mice were anesthetized with sodium pentobarbital (30 mg/kg intraperitoneally), and the portal vein was cannulated under aseptic conditions. The liver was perfused with ethylene glycol-bis (2-aminoethylether)-N,N,N',N'-tetraacetic acid (EGTA) solution (5.4 mmol/l KCl, 0.44 mmol/l KH_2_PO_4_, 140 mmol/l NaCI, 0.34 mmol/l Na_2_HPO_4_, 0.5 mmol/l EGTA, 25 mmol/l Tricine, pH 7.2) and Hank’s Balanced Salt Solution (HBSS) containing 0.075% collagenase type I (Sigma-Aldrich), 10 mg/ml DNase I (Sigma-Aldrich), 200 units/ml penicillin, and 200 μg/ml streptomycin, and then digested with 0.025% collagenase solution for the mouse liver. The isolated mouse hepatocytes were then cultured at 80%-90% confluence in DMEM medium containing 10% FBS in rat-tail collagen type I-coated 6-well plates (BD Biosciences) overnight.

**Measurement of plasma alanine aminotransferase (ALT and AST) levels—**Measurement of plasma ALT levels was performed using Alanine and Aspartate Transaminase Determination Kits purchased from Shensuo UNF (Shanghai, China) according to manufacturer’s instructions.

**Measurement of plasma triglyceride and cholesterol levels—**Measurement of plasma or liver triglyceride and cholesterol levels was performed using triglyceride kit (cat. A110-1-1) and total cholesterol kit (cat. A111-1-1) obtained from Nanjing Jiancheng Biotechnology Institute (Nanjing, China) according to manufacturer’s instructions.

**Measurement of plasma FGF21 levels—**Plasma FGF21 levels were measured using the QuantikineMouse FGF21 ELISA kit (R&D Systems)[^3^](#_ENREF_3) with a minimumdetectable dose of 3.81 pg/mL, according to themanufacturer’s instructions. Mouse plasma samples (50 μL) were mixed with 50 μL of assay diluent in a 96-well plate, and the reactions were incubated on a plate shaker at roomtemperature for 2 hours. After washing the plate 5 times, 100 μL of mouse FGF21 conjugate was added to each well, and the reactions were incubated at room temperature for 2 hours. After washing the plate 5 times, 100 μL of substrate solution was added into each well, followed by plate incubation at room temperature for 30 minutes in the dark. Once 100 μL of stop reaction solution was added to each well, the optical density at 450 nm was measured in a microplate reader (Infinite M1000; Tecan, Männedorf, Switzerland). Plasma FGF21 concentrations were calculated according to the standard curve.

**BODIPY Staining—**Hepatocytes were first fixed with 4% paraformaldehyde followed by incubation with 1:200 BODIPY® 493/503 (Thermo Fisher Scientific, Waltham, MA) for lipid staining. Cell nuclei were counterstained with DAPI (Invitrogen). Fluorescence confocal microscopy was performed using an a confocal microscope (Confocal Zeiss LSM880) using a 63x, 40x, 20x or 5x lens. ImageJ (NIH) was used to analyze the raw images. Images used in the figures are representative of trends observed in all the images obtained. More than eight representative pictures in each group were selected for quantification and statistical analysis.

**Immunoblots****—**Immunoblotting analysis was carried out as described previously[^1^](#_ENREF_1)^,^ [^3^](#_ENREF_3). In brief, mouse liver tissues or cultured cells were homogenized and lysed at 4°C in NP-40 lysis buffer (50 mM Tris-HCl, pH 8.0, 1% (v/v) Nonidet P-40, 150 mM NaCl, 5 mM EDTA, 1 mM EGTA, 1 mM sodium orthovanadate, 10 mM sodium fluoride, 1 mM phenylmethylsulfonyl fluoride, 2 μg/ml aprotinin, 5 μg/ml leupeptin, and 1 μg/ml pepstatin). For immunoblotting, 20-50 μg of protein extracts were separated by sodium dodecyl sulfate-polyacrylamide gel electrophoresis (SDS-PAGE), and then transferred to polyvinylidene difluoride (PVDF) membranes. The membranes were blocked with 5% non-fat milk in Tris-buffered saline with 0.1% tween 20 (TBST) and incubated with specific antibodies, followed by incubation with horseradish peroxidase-conjugated secondary antibodies. Immunoblots were visualized by LumiGLO chemiluminescence detection kit (Cell Signaling Technology).

**Immunohistochemistry—**Immunohistochemistry of liver sections was performed as described previously[^1^](#_ENREF_1)^,^ [^2^](#_ENREF_2). Briefly, antigen retrieval was performed, deparaffinized tissue sections were treated with 10 mmol/L citrate buffer (PH 6.0) in a microwave (2 minutes at 700W, repeated 3 times). Tissue sections were blocked with 5% normal BSA (A7906, Sigma) in phosphate-buffered saline (PBS) for 30 min. Liver sections were incubated with polyclonal antibodies against Anti-4-HNE (MHN100P, JaICA), Anti-MDA (MMD030, JaICA)[^7^](#_ENREF_7), Anti-Myeloperoxidase (GB11224, Servicebio) or Anti-F4/80 (GB113373, Servicebio) in PBS with 1% BSA overnight at 4°C and then incubated for 1 hour at room temperature with a biotinylated anti-rabbit or anti-mouse IgG secondary antibody (dilution of 1:200) using Diaminobenzidine (DAB) histochemistry Kit. Positive cells and positive areas in 5 to 10 randomly selected high-power fields were analyzed[^8^](#_ENREF_8).

**Total RNA isolation and** **Real-Time PCR—**Liver tissues and hepatocytes were homogenized in TRIzol Reagent (Life Technologies) to extract total RNAs according to the manufacturer's protocol. Total RNAs were then reversely transcribed to cDNA using SuperScript II reverse transcriptase (Life Technologies) and Oligo d (T). The resulting cDNA was subjected to real-time PCR with gene-specific primers in the presence of SYBR Green PCR master mix (Applied Biosystems) using Step One Plus Real-Time PCR System (Applied Biosystems) as described previously[^9^](#_ENREF_9). The specificity of the PCR amplification was verified by analyzing the melting curve, and also by running products on an agarose gel. Data were analyzed using the ΔΔCT threshold cycle method. mRNA levels of genes were normalized to those of GAPDH and presented as relative levels to control.

**Table S1 Concentrations of the non-ethanol ingredients determined in distilled liquors**

| Chemistry | Molecular Formula | Concentrations (mg/ L in 100% ethanol) | | | | | |
| --- | --- | --- | --- | --- | --- | --- | --- |
|  |  | Whisky | Brandy | Baijiu | Rum | Vodka |  |
| Ethyl formate | C_3_H_6_O_2_ | 14.47±0.67 | 36.09±9.62 | 14±19.8 | 1.18±1.67 | N/D |  |
| Ethyl acetate | C_4_H_8_O_2_ | 457.4±58.68 | 501.57±124 | 3757±2249.88 | 84.44±69.23 | N/D |  |
| Ethyl lactate | C_5_H_10_O_3_ | 26.74±15.05 | 104.57±14.17 | 1469.64±727.18 | N/D | N/D |  |
| Ethyl butanoate | C_6_H_12_O_2_ | N/D | N/D | 276.32±261.71 | N/D | N/D |  |
| Ethyl 2-methylbutanoate | C_7_H_14_O_2_ | N/D | N/D | 0.93±1.32 | N/D | N/D |  |
| 3-methylbutyl acetate | C_7_H_14_O_2_ | 8.39±0.95 | 3.06±0.31 | 3.21±4.54 | N/D | N/D |  |
| Ethyl pentanoate | C_7_H_14_O_2_ | N/D | N/D | 34.43±0.53 | 0.1±0.14 | N/D |  |
| Ethyl Hexanoate | C_8_H_16_O_2_ | N/D | 3.74±1.17 | 1745.7±2199.72 | N/D | N/D |  |
| Ethyl heptanoate | C_9_H_18_O_2_ | N/D | 27.61±16.17 | 8.9±6.67 | N/D | N/D |  |
| Ethyl benzoate | C_9_H_10_O_2_ | 29.43±12.73 | N/D | 6.86±9.7 | N/D | N/D |  |
| Ethyl octanoate | C_10_H_20_O_2_ | 10.11±2.47 | 29.91±1.1 | 4.45±3.2 | N/D | N/D |  |
| Ethyl 2-phenylacetate | C_10_H_12_O_2_ | N/D | 16.23±1.02 | 3.9±5.51 | N/D | N/D |  |
| 2-phenylethyl acetate | C_10_H_12_O_2_ | N/D | 10.15±0.29 | N/D | N/D | N/D |  |
| Ethyl nonanoate | C_11_H_22_O_2_ | N/D | 4.34±0.13 | 4.45±3.2 | N/D | N/D |  |
| Ethyl 3-phenylpropanoate | C_11_H_14_O_2_ | N/D | N/D | 3.44±4.87 | N/D | N/D |  |
| Ethyl decanoate | C_12_H_24_O_2_ | N/D | 6.91±0.52 | 3.4±4.8 | N/D | N/D |  |
| Ethyl tetradecanoate | C_16_H_32_O_2_ | N/D | 16.79±3.19 | 2.5±3.54 | N/D | N/D |  |
| Acetic acid | C_2_H_4_O_2_ | 333.17±17.9 | N/D | 2277.26±1625.02 | N/D | N/D |  |
| Lactic acid | C_3_H_6_O_3_ | N/D | 267.08±19.17 | 2186.77±2259.42 | N/D | N/D |  |
| Propionic acid | C_3_H_6_O_2_ | N/D | 26.07±1.71 | 93.3±89.5 | N/D | N/D |  |
| Butyric Acid | C_4_H_8_O_2_ | N/D | 59.92±13.81 | 176.54±71.66 | N/D | N/D |  |
| 2-methylpropanoic acid | C_4_H_8_O_2_ | 12.57±17.78 | 15.46±0.73 | 27.51±1.87 | N/D | N/D |  |
| Pentanoic acid | C_5_H_10_O_2_ | N/D | 24.86±0.04 | 32.77±0.49 | N/D | N/D |  |
| 3-methylbutanoic acid | C_5_H_10_O_2_ | 22.96±32.47 | 47.01±3.28 | 43.63±24.06 | N/D | N/D |  |
| Hexanoic acid | C_6_H_12_O_2_ | N/D | 27.84±0.66 | 692.06±864.84 | N/D | N/D |  |
| Heptanoic acid | C_7_H_14_O_2_ | N/D | 23.34±0.39 | 10.8±15.27 | N/D | N/D |  |
| Octanoic acid | C_8_H_16_O_2_ | 25.87±4.3 | 63.92±11.26 | 8.62±12.18 | N/D | N/D |  |
| Decanoic acid | C_10_H_20_O_2_ | 35.61±3.79 | 63.17±8.76 | N/D | N/D | N/D |  |
| Methanol | CH_4_O | 49.91±11.18 | 267.37±29.98 | N/D | 6.13±8.66 | N/D |  |
| 1-Propanol | C_3_H_8_O | 486.29±55.44 | 270.22±26.74 | 1192.78±1606.4 | 38.25±48.96 | N/D |  |
| 2-Methyl-1-propanol | C_4_H_10_O | 762.24±134.7 | 1145.69±265.66 | 143.8±192.83 | 39.78±37.57 | 0.19±0.28 |  |
| 2,3-Butanediol | C_4_H_10_O_2_ | 74.52±105.39 | 54.06±76.45 | 210.36±297.49 | N/D | N/D |  |
| 3-Methyl-1-butanol | C_5_H_12_O | 1744.78±1068.15 | 3227.3±504.01 | 1878.69±1994.94 | 126.98±116.32 | 0.84±1.19 |  |
| 1-Pentanol | C_5_H_12_O | 2.69±1.05 | 2.73±0.58 | 9.4±9.41 | 0.61±0.87 | N/D |  |
| 2-Octanol | C_8_H_18_O | N/D | 30.33±5.25 | N/D | N/D | N/D |  |
| 2-phenylethanol | C_8_H_10_O | 45.8±15.51 | 27.68±5.86 | 22.39±16.49 | N/D | N/D |  |
| Acetaldehyde | C_2_H_4_O | 178.28±36.63 | 176.07±91.16 | 1723.64±1452.93 | 134.21±189.8 | N/D |  |
| Acetoin | C_4_H_8_O_2_ | 13.55±19.16 | 27.99±0.47 | 76.65±67.12 | N/D | N/D |  |
| Furfural | C5H4O2 | 22.26±31.48 | N/D | 159.2±147.83 | N/D | N/D |  |

All values are means (mg/L in 100% ethanol) ± standard deviation (SD).

N/D: not detected.

**Table S2. The type, alcohol content and year of distilled liquors**

| **Distilled spirits** | **Whisky** | | **Brandy** | | **Baijiu** | | **Vodka** | | **Rum** | |
| --- | --- | --- | --- | --- | --- | --- | --- | --- | --- | --- |
|  | **#1** | **#2** | **#1** | **#2** | **#1** | **#2** | **#1** | **#2** | **#1** | **#2** |
| **Type** | Blended whisky | Single Malt Scotch whisky | XO Brandy | VSOP Brandy | Strong aroma Baijiu | Sauce aroma Baijiu | Neutral Vodka | Neutral  Vodka | Silver Rum | Silver Rum |
| **Alcohol content（%）** | 40 | 40 | 40 | 40 | 55 | 53 | 40 | 40 | 37.5 | 40 |
| **Year** | 12 | 12 | 10 | 4 | 2 | 2 | 1 | 1 | 1 | 1 |

**Table S3. Quantitative Real-Time PCR primers**

| **Gene** | **Species** | **Forward primer** | **Reverse primer** |
| --- | --- | --- | --- |
| GAPDH | mouse | CATCACTGCCACCCAGAAGACTG | ATGCCAGTGAGCTTCCCGTTCAG |
| IL-1β | mouse | TTAGTCCTCGGCCAAGACAG | GGCAAGGAGGAAAACACAGG |
| IL-6 | mouse | AGTTGCCTTCTTGGGACTGA | TCCACGATTTCCCAGAGAAC |
| MCP1 | mouse | CAGCCAGATGCAGTTAACGC | GCCTACTCATTGGGATCATCTTG |
| CD11b | mouse | GGCTCCGGTAGCATCAACAA | ATCTTGGGCTAGGGTTTCTCT |
| ICAM1 | mouse | GGAGCAAGACTGTGAACACG | GAGAACCACTGCTAGTCCAC |
| SREBP-1c | mouse | CCATCGACTACATCCGCTTC | GCCCTCCATAGACACATCTG |
| ACC1 | mouse | AGTGGAGCTAGAATTGGACTTG | ACAGTGGACAGAATTGAGGG |
| FAS | mouse | GCTGCGGAAACTTCAGGAAAT | AGAGACGTGTCACTCCTGGACTT |
| SCD1 | mouse | CTGTACGGGATCATACTGGTTC | CGTGCCTTGTAAGTTCTGTG |
| DGAT1 | mouse | ACCTGGCCACAATCATCTG | TGGAGTATGATGCCAGAGCA |
| ASCL4 | mouse | TTGGCTACTTACCTTTGGCTC | AATCACCCTTGCTTCCCTTC |
| FGF21 | mouse | CTGGGGGTCTACCAAGCATA | CACCCAGGATTTGAATGACC |
| SIRT1 | mouse | TCTCCTGTGGGATTCCTGAC | AAACATGGCTTGAGGGTCTG |
| CPT1a | mouse | CCAGGCTACAGTGGGACATT | GAACTTGCCCATGTCCTTGT |
| MCAD | mouse | GATCGCAATGGGTGCTTTTGATAGAA | AGCTGATTGGCAATGTCTCCAGCAAA |
| CYP2A5 | mouse | TGGTCCTGTATTCACCATCTACC | ACTACGCCATAGCCTTTGAAAA |
| OSGIN1 | mouse | CTCTCTGGACACATCCCCTAC | GAAAGGTACTCTAGGTCCTGGT |
| G6PC | mouse | CGACTCGCTATCTCCAAGTGA | GGGCGTTGTCCAAACAGAAT |
| CYP7A1 | mouse | GCTGTGGTAGTGAGCTGTTG | GTTGTCCAAAGGAGGTTCACC |
| ACOT3 | mouse | GTCGGGGTCCTTGGCATTT | GCCGATGTTGGATATAGAGCCAT |
| FABP5 | mouse | AAAGAGCTAGGAGTAGGACTGG | TGTTGCCATCACACGTAATGA |

**Reference**

1. Li Y, Xu S, Mihaylova MM, Zheng B, Hou X, Jiang B, Park O, Luo Z, Lefai E, Shyy JY, Gao B, Wierzbicki M, Verbeuren TJ, Shaw RJ, Cohen RA and Zang M. AMPK phosphorylates and inhibits SREBP activity to attenuate hepatic steatosis and atherosclerosis in diet-induced insulin-resistant mice. *Cell metabolism*. 2011;13:376-388.

2. Ma F, Liu Y, Hu Z, Xue Y, Liu Z, Cai G, Su W, Zheng Z, Fang X, Yan X, Ding D, Sun X, Jiang Y, Wei S, Li W, Zhao J, Zhang H, Li H, Xiao D, Zhang C, Ying H, Qin J, Gao X, Dai X, Fu W, Xu Y, Li Y and Cui A. Intrahepatic osteopontin signaling by CREBZF defines a checkpoint for steatosis-to-NASH progression. *Hepatology (Baltimore, Md)*. 2023;78:1492-1505.

3. Li Y, Wong K, Giles A, Jiang J, Lee JW, Adams AC, Kharitonenkov A, Yang Q, Gao B, Guarente L and Zang M. Hepatic SIRT1 attenuates hepatic steatosis and controls energy balance in mice by inducing fibroblast growth factor 21. *Gastroenterology*. 2014;146:539-49 e7.

4. Gong Q, Hu Z, Zhang F, Cui A, Chen X, Jiang H, Gao J, Chen X, Han Y, Liang Q, Ye D, Shi L, Chin YE, Wang Y, Xiao H, Guo F, Liu Y, Zang M, Xu A and Li Y. Fibroblast growth factor 21 improves hepatic insulin sensitivity by inhibiting mammalian target of rapamycin complex 1 in mice. *Hepatology*. 2016;64:425-438.

5. Zhang F, Hu Z, Li G, Huo S, Ma F, Cui A, Xue Y, Han Y, Gong Q, Gao J, Bian H, Meng Z, Wu H, Long G, Tan Y, Zhang Y, Lin X, Gao X, Xu A and Li Y. Hepatic CREBZF couples insulin to lipogenesis by inhibiting insig activity and contributes to hepatic steatosis in diet-induced insulin-resistant mice. *Hepatology*. 2018;68:1361-1375.

6. Hu Z, Han Y, Liu Y, Zhao Z, Ma F, Cui A, Zhang F, Liu Z, Xue Y, Bai J, Wu H, Bian H, Chin YE, Yu Y, Meng Z, Wang H, Liu Y, Fan J, Gao X, Chen Y and Li Y. CREBZF as a Key Regulator of STAT3 Pathway in the Control of Liver Regeneration in Mice. *Hepatology*. 2020;71:1421-1436.

7. Ma J, Guillot A, Yang Z, Mackowiak B, Hwang S, Park O, Peiffer BJ, Ahmadi AR, Melo L, Kusumanchi P, Huda N, Saxena R, He Y, Guan Y, Feng D, Sancho-Bru P, Zang M, Cameron AM, Bataller R, Tacke F, Sun Z, Liangpunsakul S and Gao B. Distinct histopathological phenotypes of severe alcoholic hepatitis suggest different mechanisms driving liver injury and failure. *The Journal of clinical investigation*. 2022;132.

8. Ma J, Guillot A, Yang ZH, Mackowiak B, Hwang S, Park O, Peiffer B, Ahmadi A, Melo L, Kusumanchi P, Huda N, Saxena R, He Y, Guan YK, Feng DC, Sancho-Bru P, Zang MW, Cameron AM, Bataller R, Tacke F, Sun ZL, Liangpunsakul S and Gao B. Distinct Histopathological Phenotypes of Severe Alcoholic Hepatitis Suggest Different Mechanisms Driving Liver Injury and Failure. *Hepatology (Baltimore, Md)*. 2022;76:S114-S114.

9. Chen X, Zhang F, Gong Q, Cui A, Zhuo S, Hu Z, Han Y, Gao J, Sun Y, Liu Z, Yang Z, Le Y, Gao X, Dong LQ, Gao X and Li Y. Hepatic ATF6 Increases Fatty Acid Oxidation to Attenuate Hepatic Steatosis in Mice Through Peroxisome Proliferator–Activated Receptor α. *Diabetes*. 2016;65:1904-1915.
